# Nucleoside Analog 2′,3′-Isopropylidene-5-Iodouridine as Novel Efficient Inhibitor of HIV-1

**DOI:** 10.3390/pharmaceutics15102389

**Published:** 2023-09-27

**Authors:** Ksenia Glumakova, Georgy Ivanov, Valeria Vedernikova, Lena Shyrokova, Timofey Lebedev, Andrei Stomakhin, Anastasia Zenchenko, Vladimir Oslovsky, Mikhail Drenichev, Vladimir Prassolov, Pavel Spirin

**Affiliations:** 1 Department of Cancer Cell Biology, Engelhardt Institute of Molecular Biology, Russian Academy of Sciences, Vavilova 32, 119991 Moscow, Russia; kglumakova@mail.ru (K.G.); georgyivanovk423@gmail.com (G.I.); vedernikova.vo@phystech.edu (V.V.); lebedevtd@gmail.com (T.L.); stomstom@hotmail.com (A.S.); kolomatchenkoa@yandex.ru (A.Z.); vladimiroslovsky@gmail.com (V.O.); mdrenichev@mail.ru (M.D.); 2 Moscow Institute of Physics and Technology, National Research University, Institutskiy per. 9, 141701 Dolgoprudny, Russia; 3 Department of Experimental Medical Science, Lund University, 221 84 Lund, Sweden; olena.shyrokova@med.lu.se; 4 Center for Precision Genome Editing and Genetic Technologies for Biomedicine, Engelhardt Institute of Molecular Biology, Russian Academy of Sciences, Vavilova 32, 119991 Moscow, Russia

**Keywords:** NRTI, nucleoside, azidothymidine, palbociclib, HIV-1, antivirals

## Abstract

Nucleoside reverse transcriptase inhibitors are the first class of drugs to be approved by the FDA for the suppression of HIV-1 and are widely used for this purpose in combination with drugs of other classes. Despite the progress in HIV-1 treatment, there is still the need to develop novel efficient antivirals. Here the efficiency of HIV-1 inhibition by a set of original 5-substituted uridine nucleosides was studied. We used the replication deficient human immunodeficiency virus (HIV-1)-based lentiviral particles and identified that among the studied compounds, 2′,3′-isopropylidene-5-iodouridine was shown to cause anti-HIV-1 activity. Importantly, no toxic action of this compound against the cells of T-cell origin was found. We determined that this compound is significantly more efficient at suppressing HIV-1 compared to Azidothymidine (AZT) when taken at the high non-toxic concentrations. We did not find any profit when using AZT in combination with 2′,3′-isopropylidene-5-iodouridine. 2′,3′-Isopropylidene-5-iodouridine acts synergistically to repress HIV-1 when combined with the CDK4/6 inhibitor Palbociclib in low non-toxic concentration. No synergistic antiviral action was detected when AZT was combined with Palbociclib. We suggest 2′,3′-isopropylidene-5-iodouridine as a novel perspective non-toxic compound that may be used for HIV-l suppression.

## 1. Introduction

Nucleoside reverse transcriptase inhibitors (NRTIs), such as Azidothymidine, Lamivudine, Didanosine, Stavudine, Abacavir, Tenofovir alafenamide, belong to the class of anti-HIV-1 drugs that are commonly used as a part of combined antiretroviral therapy (cART) [1,2,3]. As cART does not lead to HIV-1 (Human immunodeficiency virus-1) elimination, patients must take drugs for the whole life [4,5]. Most of the NRTIs used for HIV-1 treatments are toxic and many patients face long-term adverse effects, including liver, renal, and neuronal toxicity [6,7,8]. Another important aspect is the resistance of various HIV-1 strains to certain NRTIs, which in some cases significantly decrease the efficiency of cART [2,9,10,11]. Therefore, interest in the novel efficient NRTIs that pose improved metabolic characteristics, increased activity, and less cytotoxicity is very high and is focused on the synthesis of novel nucleoside inhibitors [2,12,13].

3′-Azido-2′,3′-dideoxythymidine (AZT), an inhibitor of the RNA directed DNA synthesis of the human immunodeficinecy virus (HIV), was the first drug commonly used in medicinal practice to treat HIV-1 [1,2]. In cells, this nucleoside is gradually phosphorylated to form 5′-triphosphate, which is incorporated into DNA and blocks viral DNA chain elongation because of the absence of 3′-hydroxyl terminus [14]. Though AZT has shown its efficiency and is still widely used in some cARTs [15], there is a greater tendency of RNA viruses toward mutagenesis, resulting in the formation of drug-resistant forms. This generates a need to develop new antiviral therapeutics and study their combined action with currently used drugs in order to increase the efficacy of antiviral therapy and decrease its toxicity [16]. One possible approach is the use of pyrimidine nucleosides. Which are structurally similar to AZT. Therefore, in this work, we have tested a series of 5-substituted 2′,3′-O-isopropylideneuridine derivatives and 2′,3′-O-isopropylidenethymidine as compounds, to study the suppression of HIV replication in cells. To evaluate their action on HIV replication, several experiments with AZT as a reference compound were performed. Drug interaction of pyrimidine isopropylidene derivatives with AZT or Palbociclib was studied as well. 5-substituted 2′,3′-O-isopropylideneuridines contain an isopropylidene blocking group at ribose *cis*-diol hydroxyls and therefore may act as DNA-chain terminators, analogously to AZT. Moreover, the combination of a lipophilic hydrocarbon blocking group and halogen atoms may increase the cell penetrability for such compounds. As the removal of the isopropylidene protective group requires strongly acidic conditions [17,18,19,20,21], the studied nucleosides can be stable at cellular pH values. All these factors determined the choice of 5-substituted 2′,3′-O-isopropylideneuridines as compounds possessing anti-HIV potential.

To study the antiviral action, we used HIV-1 based replication-defective lentiviral particles encoding marker eGFP. These vectors are widely used to deliver genes of interest and sequences encoding cytotoxic products or gene editing constructs [22,23,24,25]. We compared its action with the well known NRTI AZT and performed the study to evaluate whether these two drugs may be used in combination to achieve more efficient HIV-1 suppression.

Drug repurposing is a promising strategy aimed at discovering new useful activities for clinically used drugs or the compounds that failed at the last stages of clinical trials [26,27]. This is why many of the drugs used to treat different diseases are currently studied as a potential antiviral agents [28,29,30,31,32,33,34,35,36,37,38]. Interestingly, a selective inhibitor of cyclin-dependent kinase 4/6, Palbociclib, was identified as an indirect repressor of HIV-1 reverse transcription [32,33,34]. Importantly, no cytotoxic action or cell proliferation suppression effect were detected for Palbociclib at the concentrations that were used in combination with 2′,3′-isopropylidene-5-iodouridine for HIV-1 suppression.

We postulate that this compound, 2′,3′-isopropylidene-5-iodouridine, is a prospective agent for use as part of cART to suppress HIV-1.

## 2. Materials and Methods

### 2.1. Chemistry

#### 2.1.1. Reagents and Technical Equipment

The solvents and materials were reagent grade and were used without additional purification. Column chromatography was performed on silica gel (Kieselgel 60 Merck, 0.040–0.063 mm). TLC was performed on Alugram SIL G/UV254 (Macherey-Nagel) with UV visualization. ^1^H and ^13^C (with complete proton decoupling) and ^19^F NMR spectra were recorded on a Bruker AMX 300 NMR instrument and are also given in Supplementary Materials. ^1^H-NMR-spectra were recorded at 300.1 MHz, ^13^C-NMR-spectra at 75.5 MHz, and ^19^F NMR-spectra at 282.4 MHz. Chemical shifts in ppm were measured relative to the residual solvent signals as internal standards (DMSO-*d_6_*, ^1^H: 2.50 ppm, ^13^C: 39.5 ppm). Spin-spin coupling constants (*J*) are given in hertz (Hz). The double-resonance technique was applied to assign the resonances. The mass spectra were acquired using a MALDI TOF mass spectrometer AB_SCIEX_4800Plus (AB Sciex, Framingham, MA, USA) equipped with an Nd:YAG laser in the linear mode. The analysis was performed using 2,5-dihydroxybenzoic acid as the matrix. Spectra were recorded for positive ions. The spectra contained both MH^+^ peaks and (M+Na)^+^ or (M+K)^+^ peaks. Uridine (1a) (CAS 58-96-8), 5-methyluridine (ribothymidine) (1b) (CAS 1463-10-1), 5-bromouridine (1d) (CAS 957-75-5), Azidothymidine (AZT) (CAS 30516-87-1), and Palbociclib (CAS 571190-30-2) were purchased from Sigma-Aldrich (sigmaaldrich.com). Preparation of 5-fluorouridine (1c) and 5-iodouridine (1e) is described in the Supplementary Materials. All synthetic procedures as well as NMR and Mass spectra of the obtained compounds **2a**–**f** are also given in the Supplementary Materials. The chemical purity of compounds was established to be higher than 97% based on NMR analysis.

#### 2.1.2. General Procedure for Preparation of 5-Substituted 2′,3′-O-Isopropylideneuridine Derivatives

To begin, 4.1 Mmol of 5-substituted uridine derivative (R=H, CH_3_, F, Br, I) was mixed with 0.078 g (0.41 mmol) of p-methylsulfonic acid monohydrate in 20 mL of dry acetone. Then, 1.36 mL of ethylorthoformate (8.2 mmol) was added and the mixture was stirred for 24 h at room temperature. The reaction was monitored by TLC (silica gel, CH_2_Cl_2_:EtOH—97:3 (%)). Then the reaction mixture was neutralized with 0.43 g (4.1 mmol) of sodium bicarbonate and left to stir for 15 min at room temperature. The precipitate was filtered. The filtrate was evaporated in vacuo. The residue was applied into a chromatographic column with silica gel. The column was washed with CH_2_Cl_2_. The product was eluted in a CH_2_Cl_2_:EtOH—97:3 (%). The fractions containing the product were combined and evaporated in vacuo and then dried in a vacuum desiccator over P_2_O_5_.

### 2.2. Biology

#### 2.2.1. Cell Cultures

In this study, we used Jurkat and CEM-ss (human T-lymphoblastic leukemia) cells cultured in RPMI-1640 medium and HEK293T (human embryonic kidney cells) cells cultured in a DMEM medium. Growth medium was supplemented with 10% FBS (fetal bovine serum), 2 mM L-glutamine, 1mM sodium pyruvate, 100 μg/mL of streptomycin, and 100 units/mL of penicillin (Gibco/Invitrogen Life technologies). Cells were cultured at 37 °C in a humidified atmosphere with 5% CO_2_.

#### 2.2.2. Pseudotyped Lentiviral Vector Particles Production

VSV-G pseudotyped replication-defective lentiviral particles with eGFP marker gene were obtained via calcium phosphate transfection of HEK293T packaging cells (ProFection**^®^** Mammalian Transfection System, Promega) [39,40]. HEK293T cells seeded in 100 mm Petri dishes at a density of 3 × 10^6^ cells per dish were incubated for 12 h. Then cells were co-transfected with 10 μg LeGO-G2 plasmid and packaging plasmids (10 μg of pMDLg/pRRE, 5 μg of pRSV-Rev, and 2 μg VSV-G). At 8 h after transfection, the medium was changed with DMEM containing 20 mM HEPES (ThermoFisher Scientific, Waltham, MA, USA). After 12 h, the supernatants containing VSV-G-pseudotyped lentiviral particles were collected, filtered through 0.22 μM filter (Millipore), and stored at −80 °C. Pseudotyped lentiviral particles were titrated using HEK293T cells seeded to 24-well plates 24 h before transduction. To estimate an intensity of GFP expression, the number of fluorescent cells was measured on a flow cytometer (BD Biosciences, Heidelberg, Germany Biosciences) 72 h after transduction. The titer was calculated using the formula: T = N*P/V, where N is the number of seeded cells, P is the proportion of transduced cells in the population, V is the amount of added supernatant containing pseudotyped particles (mL), and T is the titer. The titer was 2.6–2.9 × 10^6^ units/mL.

#### 2.2.3. Cell Viability Analysis

**Figure 1:**
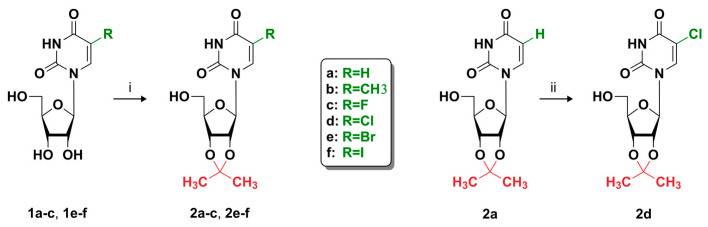
Scheme of synthesis of 5-substituted 2′,3′-O-isopropylideneuridine derivatives (**2a**–**f**). Reagents and conditions: (i) Acetone, HC(EtO)_3_, p-Me-SO_3_×H_2_O, r.t., 24 h; (ii) Nucleoside (**2a**), NClS, Py, RT, 15 min.

To assess the cytotoxic effect of AZT, nucleoside inhibitors (**2a**–**f** (Figure 1)) and Palbociclib, Jurkat, and CEM-ss cells were plated into 48-well plates (5000 cells per well) and treated with nucleoside inhibitors in the concentration range 0–30 μM or Palbociclib in the concentration range 0–6 μM. AZT and **2a**–**f** were dissolved in DMSO. The maximum concentrations of 30 μM of the studied drugs were used to exclude the possible side effects of DMSO, concentrations of which were not more than 0.03%. The equivalent concentration of DMSO (0.03%) was used in control samples of cells not treated with drugs. Palbociclib was dissolved in H_2_O. At 72 h after treatment, cell viability was assessed by staining with a 0.4% Trypan blue solution (Invitrogene Corp., Carlsbad, CA, USA) by counting in a Neubauer chamber. CC_50_s were calculated by nonlinear regression with variable slope (four parameters) and robust fitting using the GraphPad Prism software v.8.4.3 (GraphPad Software, San Diego, CA, USA).

#### 2.2.4. Analysis of Apoptosis and Cell Cycle

To evaluate the effect of Palbociclib on the apoptosis induction and on the disruption of cell cycle, Jurkat cells were plated into 48-well plates (5000 cells per well) and treated with 0, 2.9, 5.9, and 11.7 nM of Palbociclib. Apoptosis was measured by double staining with Annexin V-FITC (Invitrogene, Thermo Scientific, Waltham, MA, USA) and propidium iodide (PI) (Sigma, Cibolo, TX, USA) 72 h after transduction. To measure the distribution of cells in the phases of the cell cycle, Jurkat cells were fixed in ethanol and stained with propidium iodide [40]. All measurements were performed on an LSR Fortessa flow cytometer (BD Biosciences, San Jose, CA, USA). Analysis of apoptosis rate was performed with FlowJo software 10.0.7 (FlowJo LLC, Ashland, OR, USA). Analysis of cell cycle rate was performed with ModFit LT software v.4.1.7 (Verity Software House, Topsham, ME, USA).

#### 2.2.5. Antiviral Activity Analysis

Antiviral activity of AZT and nucleoside (**2a**–**f** (Figure 1)) dissolved in DMSO was studied using the Jurkat and CEM-ss cells seeded in 48-well plates (5000 cells per well) treated with nucleoside inhibitors in the concentration range 0–30 μM. The maximum concentrations of 30 μM of the studied drugs were used to exclude the possible side effects of DMSO, concentrations of which were not more than 0.03%. The equivalent concentration of DMSO (0.03%) was used in control samples of cells not treated with drugs. Cells were transduced with pseudotyped lentiviral particles to achieve a 50% transduction rate. At 72 h after transduction, the percentage of eGFP-positive cells was determined by flow cytometry (LSR Fortessa, BD Biosciences, Heidelberg, Germany). The results were analyzed using the FlowJo X software v10.0.7 (True Star, 448 Oregon, Chicago, IL, USA). EC_50_s were calculated by nonlinear regression with variable slope (four parameters) and robust fitting using the GraphPad Prism software v.8.4.3 (GraphPad Software, San Diego, CA, USA).

#### 2.2.6. Drug Combination Analysis

To calculate the drug combination responses, Jurkat and CEM-ss cells seeded in 96-well plates in concentration of 5000 cells per well and treated with AZT/**2f** in the concentration range 0–30 μM and Palbociclib in concentrations 0, 2.9, 5.9, and 11.7 nM both alone and in combination. The maximal concentrations of each drug (AZT/**2f**) used in combination were 30 μM. In our experiments, we decided not to exceed the concentration of 30 μM of each drug in order to exclude the possible side effects of DMSO; concentration in a well for these experiments was not more than 0.06%. The equivalent concentration of DMSO (0.06%) was used in control samples of cells not treated with drugs. A combination of a nucleoside inhibitor with Palbociclib cells were pre-treated with Palbociclib for 12 h. Then, cells were treated with AZT/**2f** and transduced with pseudotyped lentiviral particles to achieve a 50% transduction rate. At 72 h after transduction, the percentage of eGFP-positive cells was determined by flow cytometry (LSR Fortessa, BD Biosciences, Heidelberg, Germany). The synergistic and antagonistic effects of AZT, **2f**, and Palbociclib were determined using the web based SynergyFinder 3.0 software (https://synergyfinder.fimm.fi (accessed on 15 July 2023)) with a ZIP (zero interaction potency) model used for the reference. Analysis of the eGFP positive cells percentage was performed with FlowJo software 10.0.7 (FlowJo LLC, Ashland, OR, USA).

### 2.3. Data and Statistical Analysis

All available data are presented as mean ± SEM. All experiments were performed in triplicate. Statistically significant differences were assumed with *p* < 0.05. Statistical analysis was performed using GraphPad Prism software v.8.4.3 (GraphPad Software, San Diego, CA, USA). One-way ANOVA method was used to compare the treated groups with a control group. The cLogP and cLogS values were calculated using Marvin 17.21.0, Chemaxon (https://www.chemaxon.com (accessed on 3 August 2023)).

## 3. Results

### 3.1. Synthesis and Characteristics of Compounds

5-substituted uridine derivatives containing an isopropylidene blocking group were chosen as alternative structurally related AZT analogs possessing increased penetrability through the cellular membrane. Despite synthetic procedures for *cis*-diol hydroxyls protection/deprotection being well-documented, the antiviral properties of such nucleosidic intermediate products have not been substantially studied, especially in novel cellular test-systems. In this work, 5-substituted 2′,3′-O-isopropylideneuridine derivatives **2a**–**f** were generally prepared by acetalizing the corresponding 5-substituted uridines with acetone by the known procedures (Figure 1): 2′,3′-O-isopropylideneuridine (**2a**) [41], 2′,3′-O-isopropylidene-5-methyluridine (**2b**) [42], 2′,3′-O-isopropylidene-5-fluorouridine (**2c**) [43], 2′,3′-O-isopropylidene-5-bromouridine (**2e**) [44], 2′,3′-O-isopropylidene-5-ioduridine (**2f**) [45], and 2′,3′-O-isopropylidene-5-chlorouridine (**2d**) were obtained by the chlorination of 2′,3′-O-isopropylideneuridine (**2a**) with *N*-chlorosuccinimide according to the procedure described in [46]. The structure and purity of the synthesized compounds were confirmed by ^1^H and ^13^C NMR spectroscopy. The presence of the fluorine atom in compound **2c** was additionally confirmed by ^19^F NMR spectroscopy. The chemical purity of compounds was established to be higher than 97% based on NMR analysis. In addition, all compounds were characterized for purity and homogeneity by mass spectrometry (MALDI). All spectral data are presented in the Supplementary Materials.

**Figure 2:**
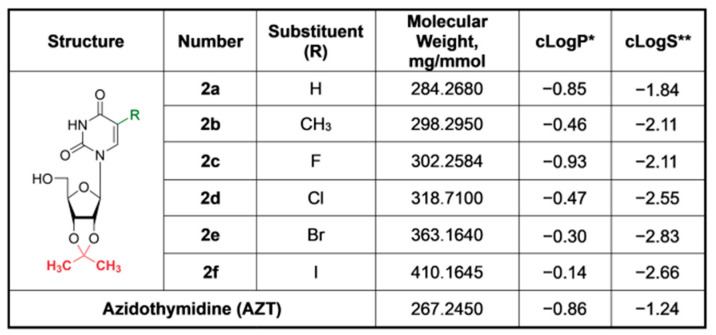
Structure and characteristics of the synthesized compounds. * The logarithm of partition coefficient between n-octanol and water log(c_octanol_/c_water_). ** Intrinsic water solubility is defined as a common solubility unit corresponding to the 10-based logarithm of the solubility of a molecule measured in mol/L after equilibrium between the dissolved and solid state at a pH where the compound is neutral). The cLogP and cLogS values were calculated using Marvin 17.21.0, Chemaxon (https://www.chemaxon.com (accessed on 3 August 2023)).

In total, a series of six compounds with substituents of different dimensions and lipophilicity at position 5 of uracil ring have been synthesized (Figure 2).

Among the synthesized 5-substituted 2′,3′-O-isopropylideneuridine derivatives, a decrease in hydrophilicity with increasing atom size (from H to I) was observed. Thus, compound **2f**, which contains a bulky iodine atom, has the lowest hydrophilicity (cLogP = −0.14) in comparison with compounds **2a**–**e** (cLogP from −0.30 to −0.93) and AZT (cLogP = −0.86) and thereby should readily penetrate the cells (Figure 2).

### 3.2. Cytotoxicity of Compounds

**Figure 3:**
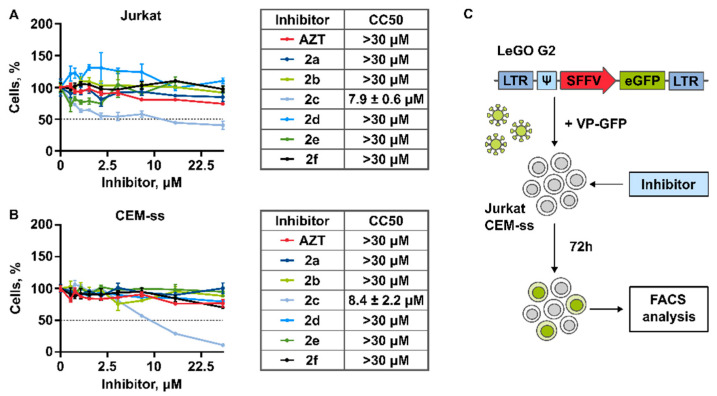
Cytotoxicity of AZT and obtained nucleoside inhibitors. (**A**,**B**) Viability of Jurkat and CEM-ss cells treated with inhibitors in the concentration range 0–30 μM represented as a percentage to non-treated control (DMSO 0.03%); dotted line shows the CC_50_ cell survival level. Right from the graphs the tables represent half-maximal cytotoxic concentrations CC_50_. (**C**) Scheme of the experiment: Jurkat and CEM-ss cells treated with AZT and obtained nucleoside inhibitors. Cells were simultaneously treated with the inhibitor and transduced with replication-defective lentiviral particles (VP-GFP) pseudotyped with VSV-G envelope protein. To obtain lentiviral particles the recombinant plasmid LeGO-G2 was used (LTR—long terminal repeats, ψ—RNA packaging signal, SFFV—spleen focus-forming virus promoter, eGFP—the sequence encoding marker fluorescent protein). The level of eGFP-positive cells was analyzed by flow cytometry 72 h after transduction.

To evaluate the antiviral activity of the obtained compounds, we used T-cells Jurkat and CEM-ss, which are widely used to study antiviral action of the potential anti-HIV-1 agents [47,48,49]. We determined that most of the studied compounds do not affect survival of Jurkat or CEM-SS cells after 72 h of incubation when taken at relatively high concentrations up to 30 μM (Figure 3A,B). The exception is the compound **2c**, which caused a significant suppressive effect on the survival of both cell lines. Within the low toxicity of the studied compounds, the half maximal cytotoxic concentrations (CC_50_) were calculated only for **2c**. Based on the data obtained, compounds **2a**, **2b**, **2d**–**2f** were chosen to study their antiviral action. It is important to note that Azidothymidine (AZT), which was used to compare the toxicity and antiviral action, did not affect cell survival at the concentrations up to 30 μM.

### 3.3. Antiviral Activity

#### 3.3.1. Generation of Lentiviral Particles Pseudotyped with VSV-G Envelope Protein

The antiviral activity of obtained compounds was studied using the model system based on replication deficient HIV-1 lentiviral particles. For the generation of lentiviral particles, the LeGo-G2 lentiviral vectors bearing the sequence encoding fluorescent protein eGFP controlled by the SFFV promoter were used [50,51,52,53] (Figure 3C). VSV-G envelope protein was used for pseudotyping of lentiviral particles. The transfer of the provirus encoding marker eGFP into the host genome provides the ability to detect the accumulation of eGFP using FACS. The efficiency of provirus synthesis is directly related to the activity of reverse transcriptase. Therefore, reverse transcriptase inhibition causes a decrease in the number of lentiviral provirus copies resulting in decrease of eGFP protein in the whole population of treated cells, which may be detected by flow cytometry [21,36]. Here, we simultaneously treated the cells with viral particles encoding eGFP (VP-GFP) and inhibitors at the wide range of concentrations up to 30 μM, followed by the FACS analysis after 72 h of incubation (Figure 3C).

**Figure 4:**
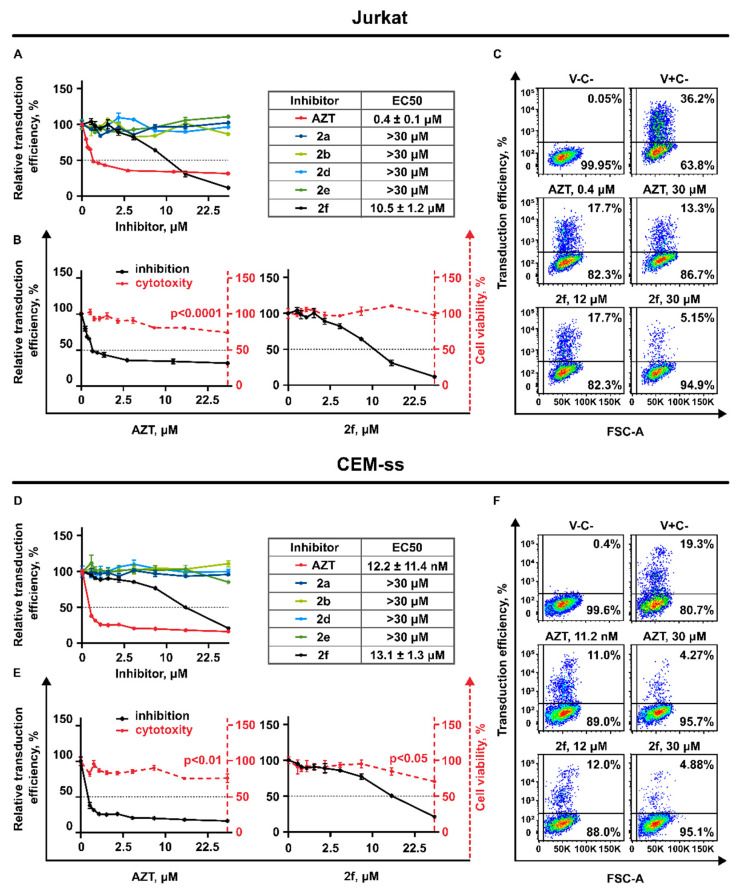
Antiviral action of AZT and the obtained nucleoside inhibitors against replication defective virus. The action of inhibitors on transduction efficiency of VP-GFP 72 h after transduction. (**A**,**D**) Jurkat and CEM-ss cells treated with inhibitors in the concentration range 0–30 μM represented as a percentage to non-treated control (DMSO 0.03%); dotted black line shows the EC_50_ level. Left from the graphs the tables representing half-maximal virus-inhibiting concentrations EC_50_. (**B**,**E**) The FACS scatter plots, represent the gating logic, changes in percentage of eGFP-positive transduced Jurkat and CEM-ss cells under treatment with AZT and **2f** at concentrations of EC_50_ and 30 μM. (V-C-)–non-transduced and not treated with inhibitor, (V+C-)—transduced but not treated with inhibitor. (**C**,**F**) The antiviral action of AZT and **2f** compared to their cytotoxic activity. Black solid lines represent the antiviral effect of AZT and **2f** added to the Jurkat and CEM-ss cells transduced with VP-GFP (left Y-axis). Red dashed lines represent the toxic effect of AZT and **2f** (left Y-axis). The dotted black horizontal line shows the EC_50_ and CC_50_ levels. Effects represented as a percentage to non-treated control (DMSO 0.03%).

Among the five studied original compounds, only one of them, **2f** (2′,3′-isopropylidene-5-iodouridine), caused the suppression of the transduction efficiency of HIV-1 lentiviral particles when added to Jurkat cells (Figure 4A,D).

To study the antiviral action and exclude the effect of multiple reinfection of target cells we used the volumes of lentiviral particles containing medium to achieve not more than 40% of eGFP positive cells for Jurkat and not more than 20% for CEM-ss cells in the whole population not treated with drugs (V+C-) (Figure 4B,E). As expected, AZT also caused significant suppressive action on VP-GFP transduction. The half maximal inhibitory concentrations were calculated for AZT and **2f**. Figure 4C,F represents the scatter plots with gating logic used for FACS analysis and the antiviral action of AZT and **2f** when the compounds were used at the CC_50_ and maximal non-toxic concentrations on 30 μM. It is important that both drugs do not suppress cell survival at the concentrations affecting transduction efficiency (Figure 4B,C,E,F). Interestingly, we evaluated that, although the anti-HIV-1 activity of AZT was significantly higher at the low concentrations (starting from 100 nM), its antiviral potential remains restricted. Starting from the concentration of 2 μM, AZT does not cause the increase of VP-GFP suppression. This seems to be the plateau effect: about 30% of Jurkat and about 20% of CEM-ss cells remains GFP positive even when AZT was used in relatively high concentration, up to 30 μM (Figure 4C,F). Importantly, compound **2f** is less efficient at the low concentrations, but the increase of its concentrations leads to significant suppression of VP-GFP, with no plateau effect as it was shown for AZT.

#### 3.3.2. AZT and **2f** Do Not Act Synergistically but Cause Pronounced Suppression of Lentiviral Transduction When Used in Combination at the High Non-Toxic Concentrations

Based on the obtained results demonstrating that the effect of AZT and **2f** on VP-GFP transduction efficiency is not the same, we decided to add both drugs simultaneously. For this we used both compounds at the wide range of concentrations. The transduction efficiency was measured and the dose-response matrixes were obtained for both cell lines Jurkat and CEM-ss (Figure S1A,B).

**Figure 5:**
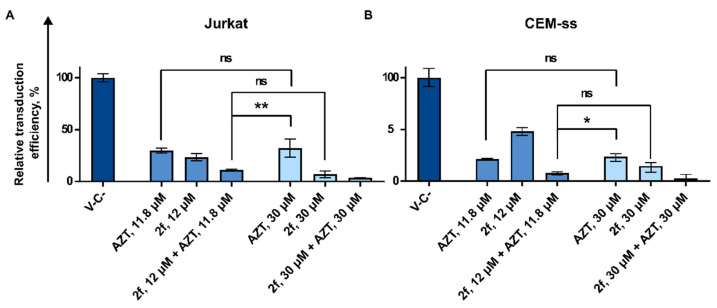
Analysis of the effect of **2f** in combination with AZT. Jurkat and CEM-ss cells were treated with AZT and **2f** in high concentrations of ~12 μM or 30 μM. Cells were simultaneously treated with the inhibitors and transduced with VP-GFP. The action of the inhibitors on transduction efficiency of VP-GFP 72 h after transduction. The graphs represent the percentage of eGFP-positive cells Jurkat (**A**) and CEM-ss (**B**) for effective combinations compared to non-treated cells (DMSO 0.06%). Asterisks: ns—*p* > 0.05, *—*p* < 0.05, **—*p* < 0.01.

Next, the synergy distribution plots were generated and analyzed using the Synergy Finder v3.0 web source. We used the ZIP model to calculate synergy scores that were used to interpret drug interaction relationships. Scores larger than 10 mean that the interaction between two drugs is synergistic. Scores up to 10 mean that the interaction is likely to be additive. The obtained results demonstrate that AZT and **2f** do not act synergistically when taken in combination (Figure S1A,B). However, we noticed that at the higher concentrations, the combination of AZT and **2f** was more effective in reducing the percentage of eGFP positive cells when compared to using them alone at the equivalent high concentrations (Figure 5A,B). As an example, when Jurkat cells were treated with VP-GFP and AZT alone at the concentration of 12 μM, this led to 70% suppression of VP-GFP transduction efficiency. Increasing the AZT concentration to 30 μM does not enhance the suppression. The compound **2f** alone acts more efficiently than AZT; increasing its concentration from 12 μM to 30 μM leads to VP-GFP suppression increasing from 70% to 90%. However, the same activity may be achieved when compound **2f**, taken at the concentration 12 μM, is supplemented with AZT at the concentration of 12 μM (Figure 5A). The similar action of the combination of AZT and **2f** used at the high but non-toxic concentrations was shown for CEM-ss cells (Figure 5B). That means that supplementing of cART, where AZT is used with **2f**, may increase the efficiency of such a therapy.

#### 3.3.3. 2′,3′-Isopropylidene-5-Iodouridine in Combination with Palbociclib Acts Synergistically to Reduce Transduction Efficiency of HIV-1 Based Replication Deficient Virus

**Figure 6:**
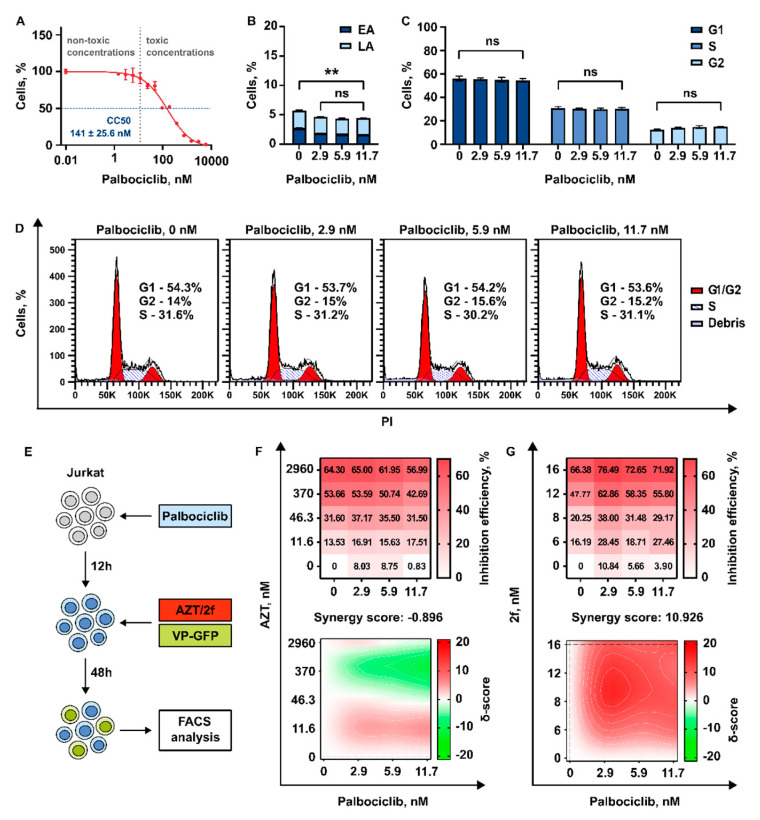
Analysis of the effect of Palbociclib in combination with nucleoside inhibitors AZT and **2f**. (**A**) Viability of Jurkat cells treated with Palbociclib in the concentration range 0–6 μM represented as a percentage to non-treated control; horizontal blue dotted line shows the CC_50_ cell survival level, vertical grey dotted line separates the ranges of toxic and non-toxic concentrations. (**B**) The graph represents a distribution of Annexin V/PI-stained Jurkat cells 72 h after treatment with non-toxic concentration of Palbociclib. (**C**,**D**) The graphs represent a distribution of PI-stained Jurkat cells 72 h after treatment with non-toxic concentration of Palbociclib by cell cycle phases. (**E**) Scheme of the experiment: Jurkat cells were pre-treated with non-toxic concentration of Palbociclib. 12 h after treatment with Palbociclib cells were treated with nucleoside inhibitor (AZT/**2f**) and transduced with VP-GFP. The level of eGFP-positive cells was analyzed by flow cytometry 72 h after transduction. (**F**,**G**) Dose-response matrix represents the percentage of eGFP-negative cells (anti-viral effect) compared to non-treated cells. Synergy plots represent the effect of the drug combination (synergism/additive effect/antagonism) calculated and visualized using SynergyFinder v3.0 software and a ZIP method. Red color highlight synergism, white—additive effect, green—antagonism. Asterisks: ns—*p* > 0.05, **—*p* < 0.01.

Palbociclib was earlier shown to upregulate SAMHD1, resulting in decrease of HIV-1 replication through depleting the intracellular pool of dNTPs [36]. We suggested that Palbociclib may increase the efficiency of modified nucleosides (NRTIs). To verify this hypothesis, we decided to use Jurkat cells. First, we determined their sensitivity to Palbociclib. The half-maximal cytotoxic concentration (CC_50_) was determined and the non-toxic range of concentrations, which may be used for the study of antiviral action, was chosen. The concentrations of Palbociclib up to 12 nM were found not to affect cell survival (Figure 6A).

Next, we evaluated that incubation with Palbociclib for 72 h at concentrations up to 12 nM does not cause the induction of apotosis in Jurkat cells (Figure 6B). Palbociclib is a classical inhibitor of cell progression and is known to induce G1 cell cycle arrest via CDK4/6 inhibition [54]. The efficiency of cell cycle progression is one of the most important factors that may affect HIV-1 replication and efficiency of HIV-1 integration into the host cell genome. Therefore, we measured how the treatment of Jurkat cell with Palbociclib in non-toxic concentrations up to 12 nM affects the cell cycle. We did not find any effect on cell cycle progression when the inhibitor was used at these concentrations (Figure 6C,D).

Subsequently, the cells were pretreated with Palbociclib for 12 h followed by an addition of VP-GFP with AZT or VP-GFP with **2f**. At 48 h post treatment with viral particles encoding marker GFP, the transduction efficiency was measured by flow cytometry (Figure 6E). In both combinations, Palbociclib was used in concentrations of ~3 nM, ~6 nM, or ~12 nM. AZT was used in three concentrations up to 3 μM, and **2f** in three concentrations up to 16 μM. The transduction efficiency was measured, and the dose-response matrixes were obtained. Notably, a very low (not more than 10%) suppressive effect of Palbociclib alone on transduction efficiency of VP-GFP was detected. Then, the dose-response matrixes were used to calculate synergy scores using ZIP algorithm (Figure 6F,G). We found that compound **2f** supplemented with Palbociclib acts synergistically to suppress transduction efficiency of HIV-1 based lentiviral particles. Interestingly, no synergistic action was detected when Palbociclib was used in combination with AZT.

## 4. Discussion

Here, we studied the antiviral activity of the 5-substituted 2′,3′-O-isopropylideneuridines, which contain an isopropylidene blocking group at ribose *cis*-diol hydroxyls. First, the cytotoxic action of these compounds was tested and we determined that most of the compounds, except of 5-fluoro-2′,3′-isopropylideneuridine, were not cytotoxic when added to Jurkat or CEM-ss cells. Importantly no cytotoxic action was evaluated when the compounds were taken, even in relatively high concentrations up to 30 μM. This is in good accordance with the data previously published by us and our colleagues [18,55,56,57]. The only compound that was found to be cytotoxic is the fluorine-containing compound. Its cytotoxic action may be explained by the properties of its fluorine atom, the dimension of which is comparable with that of a hydrogen atom. At the same time, it possesses high electronegativity. Therefore, replacement of hydrogen with fluorine leads to minor steric alterations but can modulate the properties of a parent molecule. Its acidity and basicity and/or attraction of fluorine with other functional groups in a parent molecule leads to conformational changes and can thereby affect the binding affinity of the compound with target protein molecules [56]. Highly electronegative fluorine atoms can possibly bind to several protein targets by formation of hydrogen bonds, which are not usual for other halogen atoms. High stability of C-F chemical bond can modulate metabolic stability, slowing down Cytochrome P450 monooxygenase catalyzed oxidation and hydrolytic metabolism of C-F intermediates, which are crucial for cell survival. Next, the antiviral activity of the drugs was examined. For that, we used the lentiviral vectors pseudotyped with the glycoprotein of the vesicular stomatitis virus (VSV-G). These vectors were previously successfully used to study the antiviral activity of different drugs, including the inhibitors preventing the interaction of HIV-1 cell surface receptors [22,23,24,25]. We measured the cytotoxicity of obtained potential anti-HIV-1 compounds and examined their anti-HIV-1 activity. We found that treatment of T-cells with one of the sensitized 5-halogenated uridine nucleosides 2′,3′-isopropylidene-5-iodouridine significantly repressed the transduction efficiency of HIV-1-based viral particles. Importantly, no cytotoxic action against the cells was detected when the compounds were taken, even in relatively high concentrations. We found that only 2′,3′- isopropylidene-5-iodouridine suppresses the transduction efficiency of HIV-1-based lentiviral particles. This may be explained by the structural features of this compound. It is likely that the presence of a bulky iodine atom in the structure of **2f** might affect the manifestation of inhibitory activity against HIV, in contrast to inactive compounds **2a**–**e**. The antiviral effect of iodine-substituted 2′,3′-isopropylideneuridine is possibly due to its increased hydrophobicity (Figure 2) and the large Van-der-Waals radius of iodine, which can potentiate its hydrophobic interactions with protein target(s) and increase cellular penetrability. On the one hand, AZT was found to be significantly more efficient at suppressing HIV-1 lentiviral particles when compared to **2f** when taken in relatively low concentrations up to 2 μM. This may be explained by the predicted relatively higher solvability of AZT, which results in the fact that higher concentrations of **2f** should be used to achieve the antiviral action comparable to AZT. On the other hand, we found that the anti-HIV-1 suppressive action of AZT is limited by the plateau effect, which was not found when the **2f** compound was used. The plateau effect caused by AZT was detected on both Jurkat and CEM-ss cell lines. This phenomenon was not previously shown and is of significant interest to study the features responsible for NRTIs antiviral action efficiency. Unfortunately, the mechanism of this effect is unclear. It may be explained by the heterogeneity of T-cell populations Jurkat and CEM-ss used in our study, where the subpopulation of these cells possibly remains less sensitive to anti-HIV-1 action of AZT. For example, the mechanisms of drug metabolism may be upregulated, or mechanisms of multiple drug resistance conducted via ABC transporters [58]. Furthermore, the impact of various cellular factors responsible for reverse transcriptase activity cannot be excluded, and it may also be a characteristic of the cell subpopulation, where the activity of reverse transcriptase is higher and the efficiency of AZT is decreased. The differences in the interplay of **2f** and AZT with the cellular components involved in the activity of provirus synthesis may vary in different subpopulations of cells and it may explain the differences in their action. It may partially explain the significantly higher anti-viral action of **2f** taken in combination with AZT when compared to their activity alone taken in equivalent concentrations. This may be a result of more effective inhibition of secondary intracellular targets associated with the antiviral action of NRTIs. In several studies, the dNTP depletion effect caused by treatment of cells with Palbociclib alone was identified [36,59]. Palbociclib is a well-known selective inhibitor of cyclin-dependent kinase 4/6. Palbociclib was identified as a potential repressor of HIV-1 reverse transcription through the control of sterile α motif and HD domain-containing protein-1 (SAMHD1) activity [36]. The hypothesis was that SAMHD1 decreases HIV-1 replication through its dNTP triphosphohydrolase activity by depleting the intracellular pool of dNTPs that restricts viral reverse transcription [37,38]. Here, we suggested that dNTP depletion caused by Palbociclib may increase the incorporation of modified nucleosides such as AZT during the reverse transcription resulting in more efficient suppression of HIV-1 replication. We did not find any suppressive action of Palbociclib on the HIV-1 deficient lentiviral particles transduction activity used in our system. However, we found that supplementing **2f** with Palbociclib increased its antiviral action in a synergistic manner. Notably, no increase in antiviral action of AZT was detected when used in combination with Palbociclib. The differences between **2f** and AZT structures may affect the efficiency of their incorporation into the synthesized provirus DNA strands. This may be one of the possible explanations of these effects.

## 5. Conclusions

Taken together, we conclude that 2′,3′-isopropylidene-5-iodouridine may be efficiently used for HIV-1 inhibition in non-toxic concentrations. This compound has preferences in its antiviral action, when compared to AZT, when taken at high but non-toxic concentrations and has a strong potential for supplementing cART. The novel effective combinations of this compound with the anti-HIV-1 drugs of other classes (NNRTIs, integrase, and protease inhibitors) may be used to suppress HIV-1 and these combinations should be found.

## Data Availability

The data presented in this study are available on request from the corresponding author.

## Supplementary Materials

The following supporting information can be downloaded at: https://www.mdpi.com/article/10.3390/pharmaceutics15102389/s1, A supplementary material file include the sections: Chemical Synthesis; NMR-spectrometry; Figure S1: Analysis of the effect of **2f** in combination with AZT.
