# Supplementary material for: Nucleoside Analog 2′,3′-Isopropylidene-5-Iodouridine as Novel Efficient Inhibitor of HIV-1
Source: Pharmaceutics. 2023 Sep 27;15(10):2389. doi: 10.3390/pharmaceutics15102389 (PMC10610023; doi:10.3390/pharmaceutics15102389)
Supplement: Supplementary file 1 [file pharmaceutics-15-02389-s001.zip › pharmaceutics-2602910-supplementary.pdf]

Supplementary material

1. Chemical Synthesis

1.1. Preparation of initial 5-substituted uridine derivatives

5-Fluorouridine (1c)

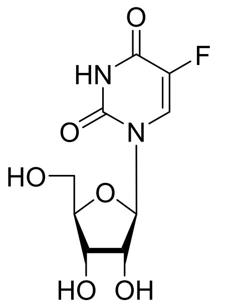

3 g (23.06 mmol) of 5-fluorouracil in a 100 mL flask and 11.01 g (34.6 mmol) of 1-O-acetyl-2,3,5-tri-O-acetyl-β-D-ribofuranose in a 100 mL drop funnel were dried in the desiccator in vacuo over P_2O_5 for 24 hours. The desiccator was filled with nitrogen. The content of the dropping funnel was dissolved in dry dichloroethane (16 mL). The content of the flask was treated with 11.3 mL (46.12 mmol) bis(trimethylsilyl)acetamide (BSA) and left to stir for 15 min at 60°C. A solution of 1-*O*-acetyl-2,3,5-tri-*O*-acetyl-β-D-ribofuranose was added to the resulting solution dropwise and 12.4 mL (69.18 mmol) trimethylsilyl trifluoromethanesulfonate was then added to the resulting mixture. The mixture was left to stir and reflux at 60°C for 1 hour, then cooled to ambient temperature, diluted with ethyl acetate (100 mL) and poured into separating funnel. The organic layer was washed with saturated aqueous sodium bicarbonate solution (150 mL), the organic layer was separated, and the aqueous layer was twice extracted with ethyl acetate (150 mL). The organic layers were combined, dried over anhydrous sodium sulfate, filtered and evaporated in vacuum. The residue was subjected to chromatographic purification on silica gel (270 mL) in a methylene chloride:ethanol mixture. The column was washed with methylene chloride. The product was eluted in a methylene chloride:ethanol (96:4, v/v) mixture. The fractions containing the product were combined and evaporated in vacuum to dryness. The yield of 2',3',5'-tri-O-acetyl-5-fluorouridine was 2.34 g (26 %) as a white foam. R_f: 0.45 (methylene chloride:ethanol - 96:4, *v/v*). ^{1}H-NMR (DMSO-d_6): 11.97 (s, 1H, N^{3}H), 8.10 (d, 1H, $J_{6\text{-}F}$=7.0, H-6 Ura), 5.88 (dd, 1H, $J_{1'\text{-}2'}$ = 5.2, $J_{1'\text{-}F}$ = 0.9, H-1'), 5.45 (dd, 1H, $J_{2'\text{-}1'}$ = 5.2, $J_{2'\text{-}3'}$=6.3, H-2'), 5.31 (dd, 1H, $J_{3'\text{-}2'}$ = 6.3, $J_{3'\text{-}4'}$ = 5.0, H-3'), 4.38-4.22 (m, 3H, H-4', H-5'a, H-5'b), 2.07 (c, 3H, Ac), 2.06 (c, 3H, Ac), 2.05 (c, 3H, Ac).

A solution of 0.904 g (2.33 mmol) of 2',3',5'-tri-O-acetyl-5-fluorouridine in 23.4 mL of 5M propylamine in methanol ($PrNH_2$/MeOH) was left to stay at ambient temperature for 12 h and then evaporated. The residue was subjected to chromatographic purification on silica gel (90 mL). The column was washed with methylene chloride. The product was eluted in a methylene chloride:ethanol (8:2, *v/v*) mixture. The fractions containing the product were combined and

evaporated to dryness. The residue was mixed with a catalytic amount of activated carbon and dissolved in 50 mL of ethanol and 30 mL of dioxane. The mixture was refluxed for 5 minutes and then filtered through celite (*Hyflo Super Cel*). The filtrate was evaporated in vacuum to dryness and precipitated from hexane. The yield 0.562 g (91%) as a white powder. R_f: 0.64 (methylene chloride:ethanol - 8:2, *v/v*). ^{1}H-NMR (DMSO-d_6): 11.68 (br s, 1H, N^{3}H), 8.28 (d, 1H, ^3J = 7.4, H-5 5-F-Ura), 5.73 (dd, 1H, $J_{1'\text{-}2'}$ = 4.4, $J_{1'\text{-}F}$ =1.8, H-1'), 5.38 (d, 1H, $J_{2'\text{-}OH}$ = 4.7, 2'-OH), 5.23 (t, 1H, $J_{5'\text{-}OH}$=4.6, 5'-OH), 5.05 (d, 1H, $J_{3'\text{-}OH}$ =3.5, 3'-OH), 4.02 (dd, 1H, $J_{2'\text{-}1'}$ = 4.4, $J_{2'\text{-}3'}$ = 4.3, $J_{2'\text{-}OH}$ = 4.7, H-2', overlapping with H-3'), 3.99-3.95 (м, 1H, H-3', overlapping with H-2'), 3.85 (ddd, 1H, $J_{4'\text{-}3'}$ = 5.5, $J_{4'\text{-}5'a}$ = 2.9, $J_{4'\text{-}5'b}$ = 2.4, H-4'), 3.68 (ddd, 1H, $J_{5'a\text{-}5'b}$=11.6, $J_{5'\text{-}OH}$=4.6, $J_{5'a\text{-}4'}$=2.9, H-5'a), 3.63 (ddd, 1H, $J_{5'a\text{-}5'b}$=11.6, $J_{5'\text{-}OH}$=4.6, $J_{5'b\text{-}4'}$=2.4, H-5'b). ^{13}C-NMR (DMSO-d_6): 156.99 (d, ^2J = 26.2, 4-*C*=O 5-F-Ura), 149.23 (2-*C*=O 5-F-Ura), 139.91 (d, ^1J = 229.9, *C*-5 5-F-Ura), 124.83 (d, ^2J = 34.7, *C*-6, 5-F-Ura), 88.24 (C1′), 84.70 (C4′), 73.75 (C2′), 69.41 (C3′), 60.37 (C5′). ^{19}F NMR (282.4 *MHz*, DMSO-*d6*): δ = −167.65.

5-Ioduridine (1f)

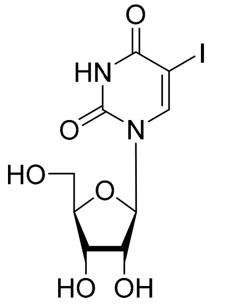

A solution of uridine (5 g, 20.5 mmol) and iodine (13.53 g, 53.3 mmol) was dissolved in 1 M aqueous nitric acid (41.8 mL) and dioxane (80 mL) was refluxed for 5 h and then evaporated in vacuum. The product was recrystallized from ethanol and dried in a vacuum desiccator over phosphorus pentoxide. Yield 4.3 g (57%) as a white powder with a slight cream color. R_f: 0.83 (methylene chloride:ethanol - 85:15, *v/v*). ^{1}H-NMR (DMSO-d_6): 11.65 (c, 1H, N^{3}H), 8.46 (c, 1H, H-6), 5.72 (d, 1H, $J_{1'\text{-}2'}$ = 4.6, H-1'), 5.36 (br s, 1H, OH), 5.22 (br s, 1H, OH), 5.03 (br s, 1H, OH), 4.03 (dd, 1H, $J_{2'\text{-}3'}$ =4.7, $J_{2'\text{-}1'}$ = 4.6, H-2'), 3.98 (t, 1H, $J_{2'\text{-}3'}$ =4.7, H-3'), 3.91 – 3.82 (m, 1H, H-4'), 3.68 (dd, 1H, $J_{5'a\text{-}5'b}$ =12.0, $J_{5'a\text{-}4'}$=2.7, H-5'a), 3.56 (dd, $J_{5'b\text{-}5'a}$ =12.0, $J_{5'b\text{-}4'}$ =2.4, H-5'b). ^{13}C-NMR (DMSO-d_6): 160.45, 150.35, 145.13, 88.31, 84.72, 73.92, 69.38, 69.24 (*C*-5, 5-I-Ura), 60.21.

1.2. Preparation of final 5-substituted 2',3'-O-isopropylideneuridine derivatives

2',3'-O-isopropylideneuridine (2a)

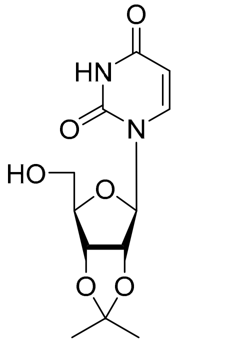

Yield 0.56 g (48%) as a white foam, Rf: 0.35 (methylene chloride:ethanol - 97:3, %). ^{1}H-NMR (DMSO-d_6): 11.38 (s, 1H, N^{3}H), 7.79 (d, 1H, $J_{6\text{-}5}$=8.1 Hz, H-6 Ura), 5.83 (d, 1H, $J_{1'\text{-}2'}$ =2.7 Hz, H-1'), 5.63 (dd, 1H, $J_{5\text{-}6}$=8.0 Hz, $J_{5\text{-}3}$=2.2 Hz, H-5 Ura), 5.08 (t, 1H, $J_{5'\text{-}OH}$ = 5.1 Hz, 5'-OH), 4.89 (dd, 1H, $J_{2'\text{-}3'}$ = 6.4 Hz, $J_{2'\text{-}1'}$ = 2.7 Hz, H-2'), 4.74 (dd, 1H, $J_{3'\text{-}2'}$ =6.4 Hz, $J_{3'\text{-}4'}$ =3.6 Hz, H-3'), 4.06 (ddd, 1H, $J_{4'\text{-}3'}$ =3.6 Hz, $J_{4'\text{-}5'b}$ =4.9 Hz, $J_{4'\text{-}5'a}$ =4.7 Hz, H-4'), 3.60 (ddd, 1H, $J_{5'a\text{-}5'b}$ = -11.8 Hz, $J_{5'\text{-}OH}$ = 5.1 Hz, $J_{5'a\text{-}4'}$ = 4.7 Hz, H-5'a), 3.54 (ddd, 1H, $J_{5'b\text{-}5'a}$ = -11.8 Hz, $J_{5'\text{-}OH}$ = 5.1 Hz, $J_{5'b\text{-}4'}$ = 4.9 Hz, H-5'b), 1.48 (s, 3H, CH_3), 1.28 (s, 3H, CH_3). ^{13}C-NMR (DMSO-d_6): 163.17 (C-4), 150.34 (C-2), 141.90 (C-6), 112.97 (<u>C</u>Me_2-isoprop), 101.73 (C-5), 91.12 (C1′), 86.51 (C4′), 83.68 (C2′), 80.48 (C3′), 61.27 (C5′), 27.04 (CH3-isoprop), 25.18 (CH3-isoprop). Mass spectrum (MALDI): m/z [M$^+$] calculated for $C_{12}H_{16}IN_2O_6$ 284.27; found 284.01.

2',3'-O-isopropylidene-5-methyluridine (2b)

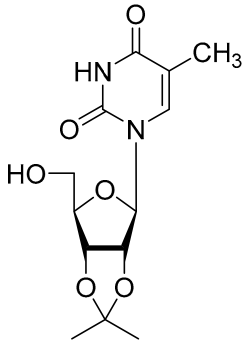

Yield 0.28 g (25%) as a white foam, Rf: 0.11 (methylene chloride:ethanol - 97:3, %). ^{1}H-NMR (DMSO-d_6): 11.34 (s, 1H, N^{3}H), 7.64 (d, 1H, $J_{6\text{-}CH3}$=8.1 Hz, H-6 Ura), 5.83 (d, 1H, $J_{1'\text{-}2'}$ =2,9 Hz, H-1'), 5.07 (t, 1H, $J_{5'\text{-}OH}$ = 5.3 Hz, 5'-OH), 4.88 (dd, 1H, $J_{2'\text{-}3'}$ = 6.4 Hz, $J_{2'\text{-}1'}$ = 2.9 Hz, H-2'), 4.75 (dd, 1H, $J_{3'\text{-}2'}$ =6.4 Hz, $J_{3'\text{-}4'}$ =3.7 Hz, H-3'), 4.03 (ddd, 1H, $J_{4'\text{-}3'}$ =3.7 Hz, $J_{4'\text{-}5'b}$ =4.9 Hz, $J_{4'\text{-}5'a}$ =4.7 Hz, H-4'), 3.60 (m, 2H, H-5'a, H-5'b), 1.77 (d, $J_{CH3\text{-}6}$=0.9 Hz, CH_3-Thy), 1.48 (s, 3H, CH_3), 1.27 (s, 3H, CH_3). ^{13}C-NMR (DMSO-d_6): 163.72 (C-4), 150.32 (C-2), 137.41 (C-6), 113.08 (<u>C</u>Me_2-isoprop), 109.44 (C-5), 90.42 (C1′), 86.07 (C4′), 83.68 (C2′), 80.36 (C3′), 61.23 (C5′), 27.02 (CH3-isoprop), 25.16 (CH3-isoprop), 12.04 (5-CH3). Mass spectrum (MALDI): m/z [M$^+$] calculated for $C_{12}H_{16}IN_2O_6$ 284.27; found 284.01.

2',3'-O-isopropylidene-5-fluorouridine (2c)

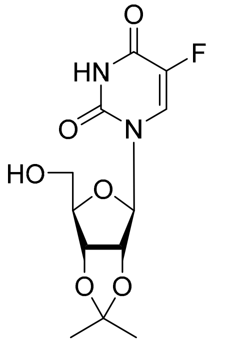

Yield 0.595 g (48%) as a white foam. Rf: 0.19 (methylene chloride:ethanol - 97:3, v/v). ^{1}H-NMR (DMSO-d_6): 11.87 (s, 1H, N^{3}H), 8.18 (d, 1H, $J_{6\text{-F}}$=7.2 Hz, H-6 Ura), 5.83 (dd, 1H, $J_{1'\text{-}2'}$ =2.5 Hz, $J_{1'\text{-F}}$ =1.1 Hz, H-1'), 5.20 (t, 1H, $J_{5'\text{-OH}}$ = 5.1 Hz, 5'-OH), 4.88 (dd, 1H, $J_{2'\text{-}3'}$ = 6.3 Hz, $J_{2'\text{-}1'}$ =2.7 Hz, H-2'), 4.76 (dd, 1H, $J_{3'\text{-}2'}$ = 6.3 Hz, $J_{3'\text{-}4'}$ =3.4 Hz, H-3'), 4.10 (ddd, 1H, $J_{3'\text{-}4'}$ = 3.4 Hz, $J_{4'\text{-}5'a}$ = 4.1 Hz, $J_{4'\text{-}5'b}$ = 4.4 Hz, H-4'), 3.66 (ddd, 1H, $J_{5'a\text{-}5'b}$ = 11.9 Hz, $J_{5'\text{-OH}}$ = 5.1 Hz, $J_{5'a\text{-}4'}$ = 4.1 Hz, H-5'a), 3.56 (ddd, 1H, $J_{5'b\text{-}5'a}$ = -11.9 Hz, $J_{5'\text{-OH}}$ = 5.1 Hz, $J_{5'b\text{-}4'}$ = 4.4 Hz, H-5'b), 1.48 (s, 3H, CH_3), 1.29 (s, 3H, CH_3). ^{13}C-NMR (DMSO-d_6): 157.01 (d, ^{2}J = 26.0 Hz, C-4), 148.93 (C-2), 139.88 (d, ^{1}J = 230.3 Hz, C-5), 125.80 (d, ^{2}J = 34.8 Hz, C-6), 112.89 (<u>C</u>Me$_2$-isoprop), 90.92 (C1′), 86.49 (C4′), 83.72 (C2′), 80.20 (C3′), 61.11 (C5′), 26.98 (CH3-isoprop), 25.14 (CH3-isoprop). ^{19}F NMR (282.4 *MHz*, DMSO-*d6*): δ = −167.65. Mass spectrum (MALDI): m/z [M$^+$+Na$^+$] calculated for $C_{12}H_{15}FN_2O_6Na^+$ 325.24; found 325.74.

2',3'-O-isopropylidene-5-bromouridine (2e)

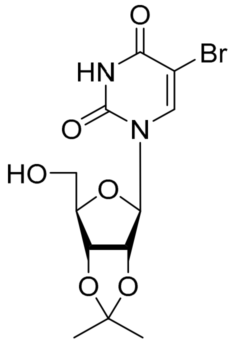

Yield 0.834 g (56%) as a white foam. Rf 0.36 (methylene chloride:ethanol - 97:3, v/v). ^{1}H-NMR (DMSO-d_6): 11.85 (s, 1H, N^{3}H), 8.33 (s, 1H, H-6 Ura), 5.83 (d, 1H, $J_{1'\text{-}2'}$ = 2.5 Hz, H-1'), 5.18 (t, 1H, $J_{5'\text{-OH}}$ =5.1 Hz, 5'-OH), 4.92 (dd, 1H, $J_{2'\text{-}3'}$ = 6.3 Hz, $J_{2'\text{-}1'}$ = 2.5 Hz, H-2'), 4.76 (dd, 1H, $J_{3'\text{-}2'}$ =6.3 Hz, $J_{3'\text{-}4'}$ = 3.5 Hz, H-3'), 4.11 (ddd, 1H, $J_{3'\text{-}4'}$ =3.5 Hz, $J_{4'\text{-}5'a}$ = 4.0 Hz, $J_{4'\text{-}5'b}$ = 4.2 Hz, H-4'), 3.63 (ddd, 1H, $J_{5'a\text{-}5'b}$ = -11.9 Hz, $J_{5'\text{-OH}}$ = 5.1 Hz, $J_{5'a\text{-}4'}$ = 4.0 Hz, H-5'a), 3.56 (ddd, 1H, $J_{5'b\text{-}5'a}$ = -11.9 Hz, $J_{5'\text{-OH}}$ = 5.1 Hz, $J_{5'b\text{-}4'}$ = 4.2 Hz, H-5'b), 1.48 (c, 3H, CH_3), 1.29 (c, 3H, CH_3). ^{13}C-NMR (DMSO-d_6): 159.15 (C-4), 149.66 (C-2), 141.30 (C-6), 112.86 (<u>C</u>Me$_2$-isoprop), 95.79 (C-5), 91.40 (C1′), 86.91 (C4′), 83.92 (C2′), 80.22 (C3′), 61.07 (C5′), 26.96 (CH3-isoprop), 25.12 (CH3-isoprop). Mass spectrum (MALDI): m/z [M$^+$+K$^+$] calculated for $C_{12}H_{15}BrN_2O_6K^+$ 402.26; found 402.19.

2',3'-O-isopropylidene-5-ioduridine (2f)

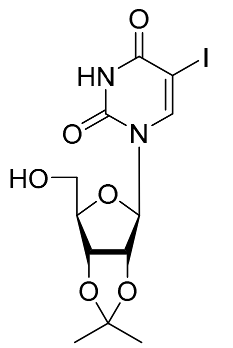

Yield 0.886 g (80%) as a white foam. Rf 0.62 (methylene chloride:ethanol - 97:3, v/v). ^{1}H-NMR (DMSO-d_6): 11.73 (s, 1H, N^{3}H), 8.32 (s, 1H, H-6), 5.82 (d, 1H, $J_{1'-2'}$ =2.4 Hz, H-1'), 5.17 (t, 1H, $J_{5'-OH}$ =5.1 Hz, 5'-OH), 4.92 (dd, 1H, $J_{2'-3'}$ = 6.3 Hz, $J_{1'-2'}$ = 2.4, H-2'), 4.75 (dd, 1H, $J_{3'-2'}$ =6.3 Hz, $J_{3'-4'}$ =3.5, H-3'), 4.09 (ddd, 1H, $J_{3'-4'}$ =3.5 Hz, $J_{4'-5'a}$ =3.9 Hz, $J_{4'-5'b}$ = 4.4 Hz, H-4'), 3.62 (ddd, 1H, $J_{5'a-5'b}$ = -11.8 Hz, $J_{5'-OH}$ = 5.1 Hz, $J_{5'a-4'}$ = 3.9 Hz, H-5'a), 3.56 (ddd, 1H, $J_{5'b-5'a}$ = -11.8 Hz, $J_{5'-OH}$ = 5.1 Hz, $J_{5'b-4'}$ =4.4 Hz, H-5'b), 1.48 (s, 3H, CH_3), 1.29 (s, 3H, CH_3).

^{13}C-NMR (DMSO-d_6): 160.55 (C-4), 150.05 (C-2), 146.11 (C-6), 112.88 (**C**Me_2-isoprop), 91.34 (C1′), 86.90 (C4′), 83.89 (C2′), 80.28 (C3′), 69.47 (C-5), 61.10 (C5′), 26.98 (CH3-isoprop), 25.14 (CH3-isoprop). Mass spectrum (MALDI): m/z [M$^+$] calculated for $C_{12}H_{14}IN_2O_6^+$ 409.16; found 409.62.

2',3'-O-isopropylidene-5-chlorouridine (2d)

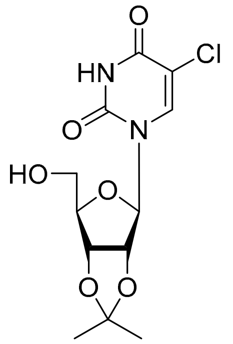

A solution of 2',3'-O-isopropylideneuridine (0.302 g, 1.06 mmol) and *N*-chlorosuccinimide (0.453 g, 3.4 mmol) in 10 mL of pyridine was held at ambient temperature for 40 min and then evaporated in vacuum. The residue was dissolved in a minimal amount of methylene chloride (~5 mL) and applied to a silica gel column for separation. The column was washed with methylene chloride. The product was eluted in a methylene chloride:ethanol mixture (97:3, *v*/*v*). The fractions containing the product were combined and evaporated to dryness. The residue was mixed with a catalytic amount of activated charcoal and dissolved in 50 mL of ethanol. The mixture was refluxed for 5 minutes, filtered through cellite (Hyflo Super Cel) and the filtrate was evaporated in vacuum to dryness and co-precipitated with hexane. Yield 0.119 g (35%) as a white foam. R_f: 0.24 (methylene chloride:ethanol - 97:3, *v*/*v*). ^{1}H-NMR (DMSO-d_6): 11.90 (s, 1H, N^{3}H), 8.26 (s, 1H, H-6 Ura), 5.83 (d, 1H, $J_{1'-2'}$ =2.5 Hz, H-1'), 5.20 (t, 1H, $J_{5'-OH}$ = 5.0 Hz, 5'-OH), 4.92 (dd, 1H, $J_{2'-3'}$ =6.3 Hz, $J_{2'-1'}$ =2.5, H-2'), 4.76 (dd, 1H, $J_{3'-2'}$ = 6.3 Hz, $J_{3'-4'}$ =3.5

Hz, H-3'), 4.11 (ddd, 1H, $J_{3'-4'}$ =3.5 Hz, $J_{4'-5'a}$ =3.9 Hz, $J_{4'-5'b}$ =4.2 Hz, H-4'), 3.63 (ddd, 1H, $J_{5'a-5'b}$ = -11.9 Hz, $J_{5'-OH}$ = 5.0 Hz, $J_{5'a-4'}$ =3.9 Hz, H-5'a), 3.56 (ddd, 1H, $J_{5'b-5'a}$ = -11.9 Hz, $J_{5'-OH}$ = 5.1 Hz, $J_{5'b-4'}$ =4.2 Hz, H-5'b), 1.48 (c, 3H, CH_3), 1.28 (c, 3H, CH_3). ^{13}C-NMR (DMSO-d_6): 159.00 (C-4), 149.44 (C-2), 138.84 (C-6), 112.87 (**C**Me2-isoprop), 107.22 (C-5), 91.34 (C1′), 86.86 (C4′), 83.91 (C2′), 80.20 (C3′), 61.06 (C5′), 26.96 (CH3-isoprop), 25.13 (CH3-isoprop). Mass spectrum (MALDI): m/z [M^{+}+ Na^{+}] calculated for $C_{12}H_{15}ClN_2O_6Na^{+}$ 341.69; found 341.31.

2. NMR-spectrometry

^{1}H and ^{13}C (with complete proton decoupling) NMR spectra were recorded on Bruker AMX 300 NMR instrument and are given in Supplementary Part. ^{1}H-NMR-spectra were recorded at 300.1 MHz, ^{13}C-NMR-spectra at 75.5 MHz and the ^{19}F-NMR-spectra at 282.4 MHz. Chemical shifts in ppm were measured relative to the re-sidual solvent signals as internal standards (DMSO-d_6, ^{1}H: 2.50 ppm, ^{13}C: 39.5 ppm). Spin-spin coupling constants (*J*) are given in Hz.

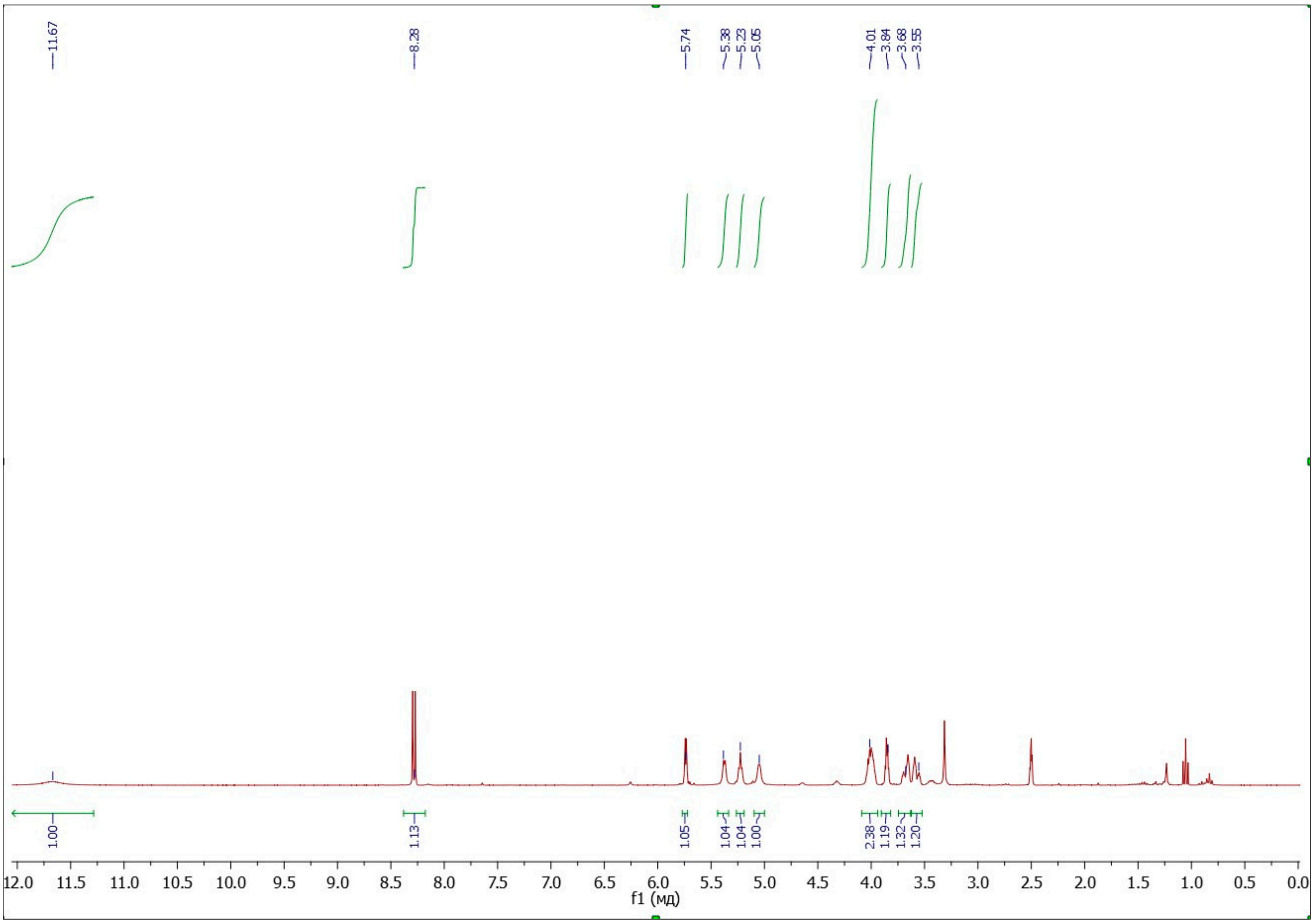

^{1}H-NMR-spectrum (300.1 MHz) of 5-Fluorouridine **(1c)** in DMSO-d_6 at 303 K

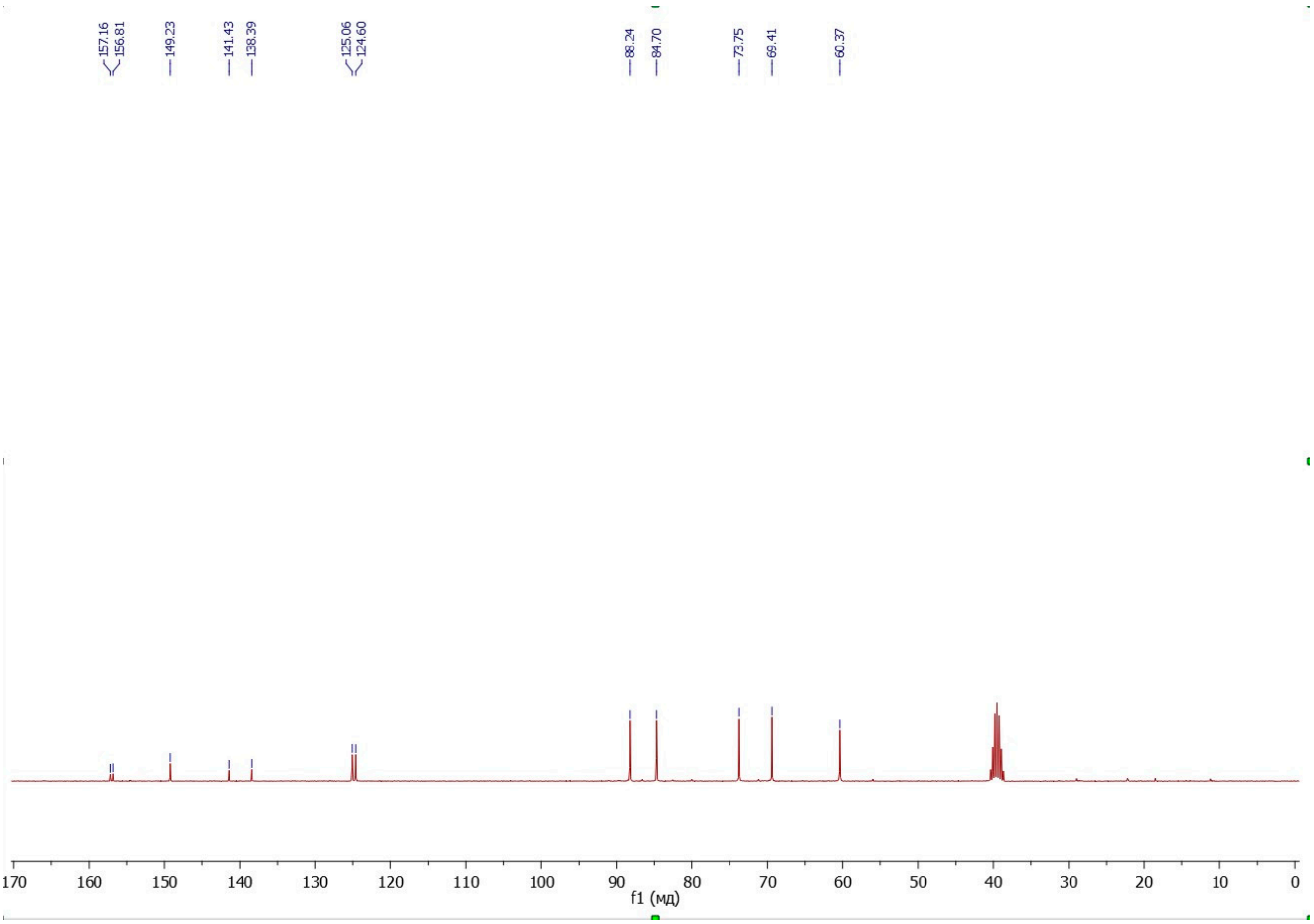

^{13}C-NMR-spectrum (75.5 MHz) of 5-Fluorouridine **(1c)** in DMSO-d_6 at 303 K

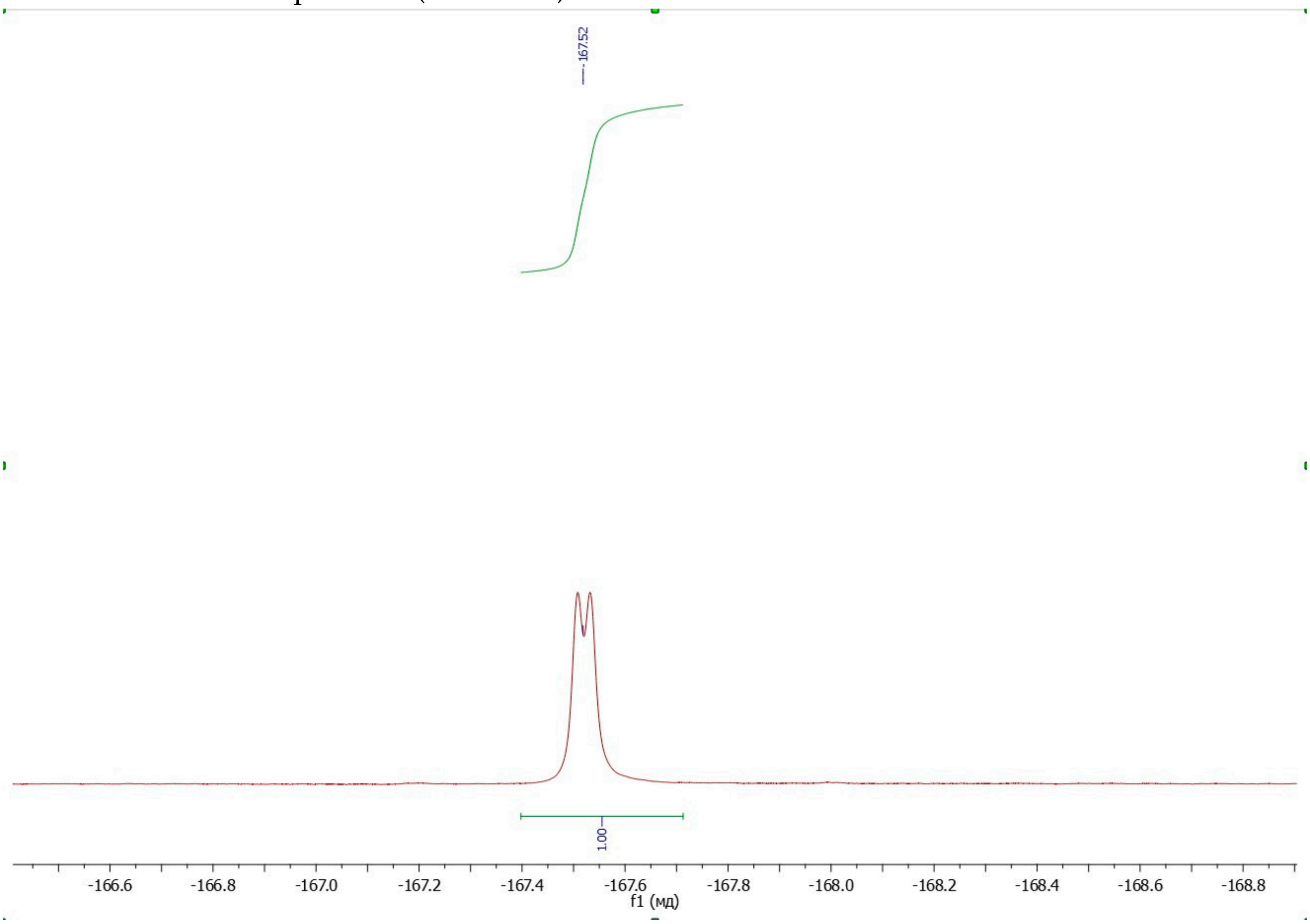

^{19}F-NMR-spectrum (282.4 MHz) of 5-Fluorouridine **(1c)** in DMSO-d_6 at 303 K

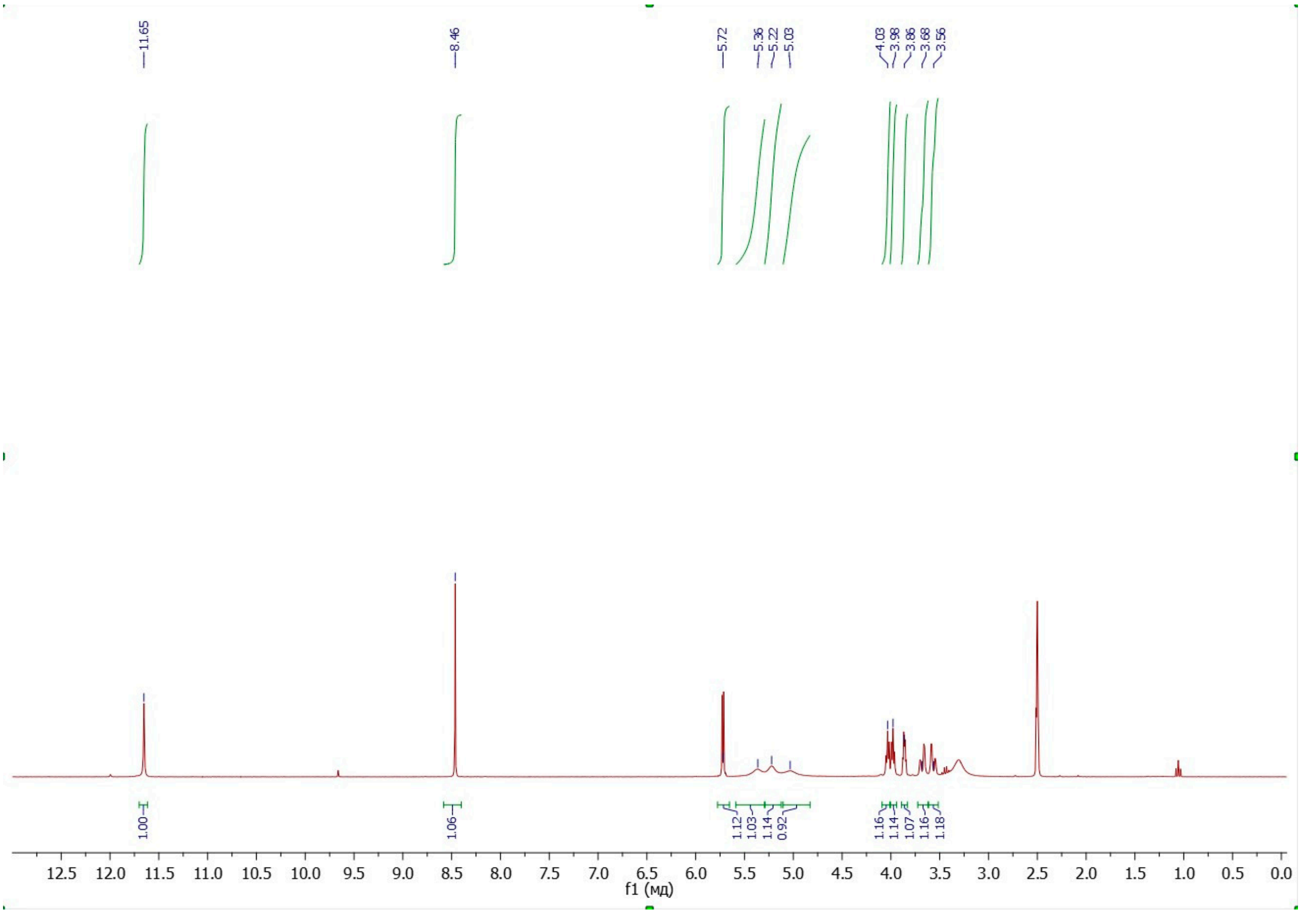

^{1}H-NMR-spectrum (300.1 MHz) of 5-Iodouridine **(1f)** in DMSO-d_6 at 303 K

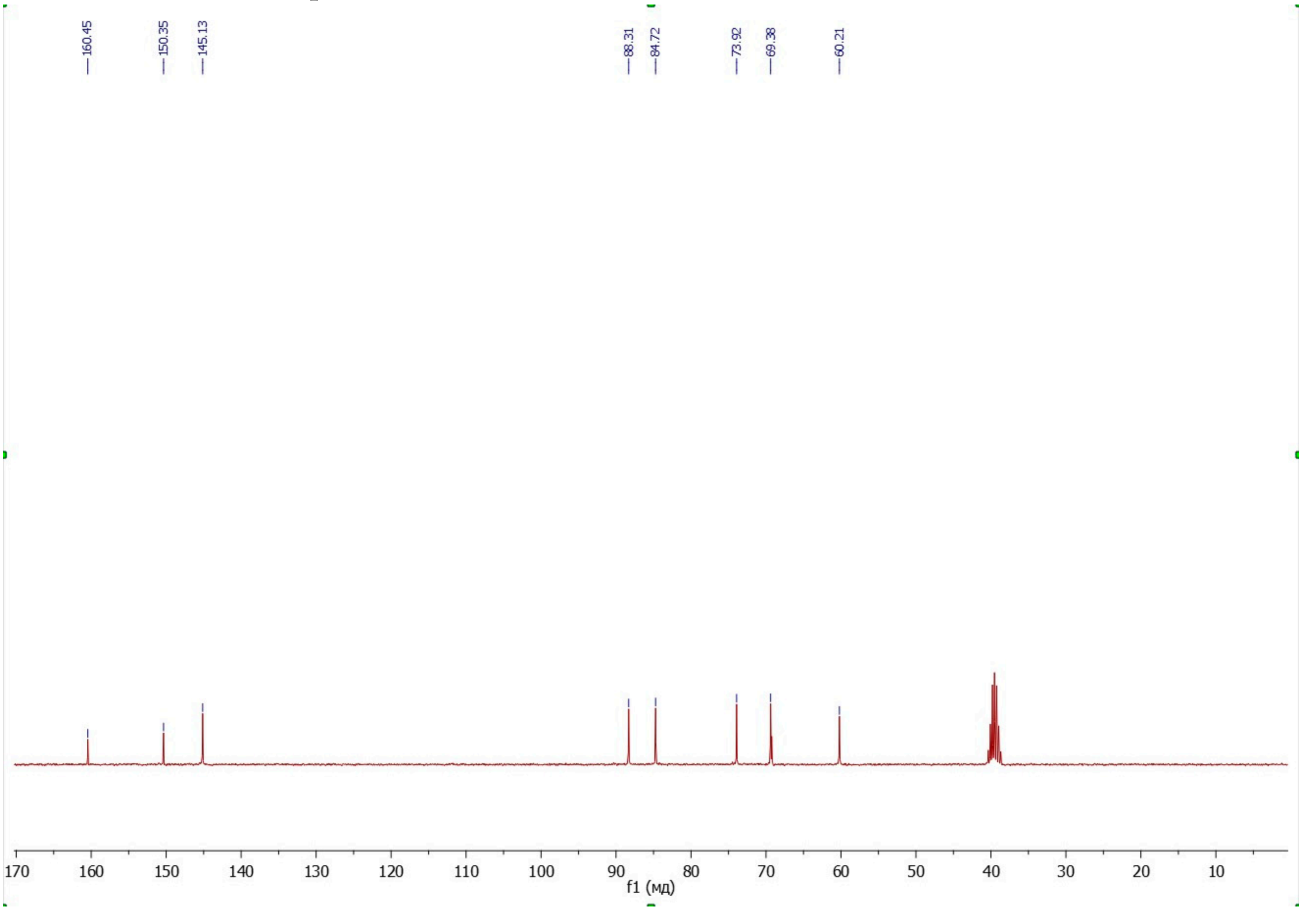

^{13}C-NMR-spectrum (75.5 MHz) of 5-Iodouridine **(1c)** in DMSO-d_6 at 303 K

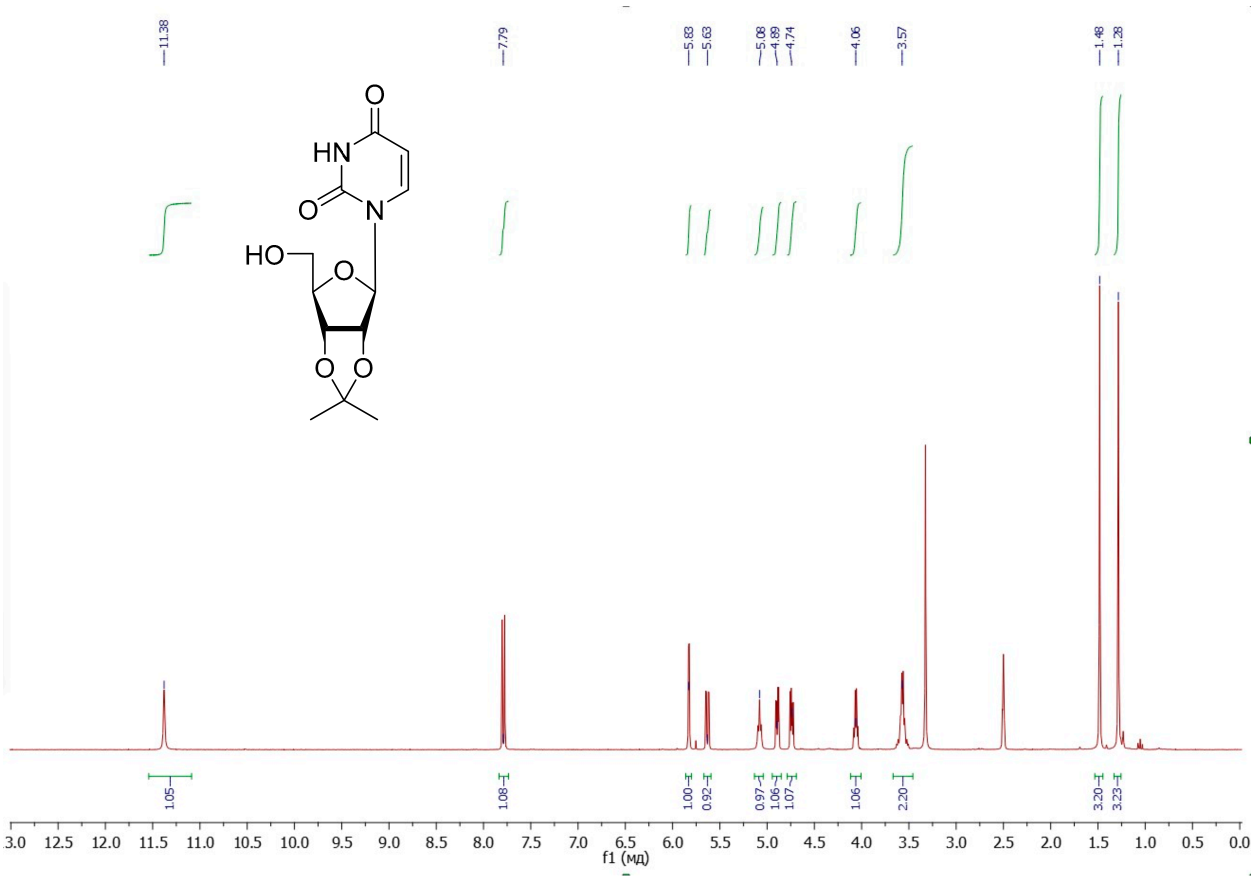

^{1}H-NMR-spectrum (300.1 MHz) of 2',3'-O-isopropylideneuridine **(2a)** in DMSO-d_6 at 303 K

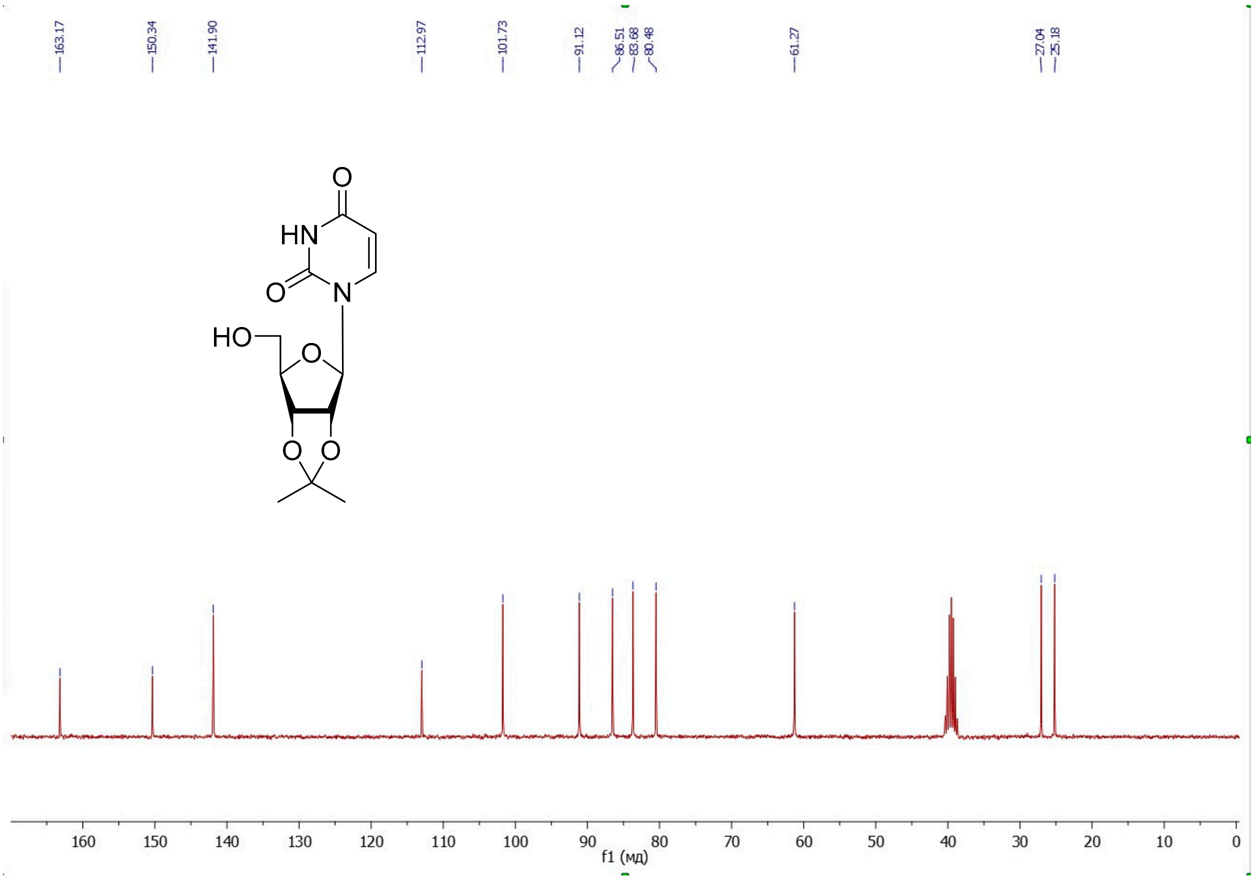

^{13}C-NMR-spectrum (75.5 MHz) of 2',3'-O-isopropylideneuridine **(2a)** in DMSO-d_6 at 303 K

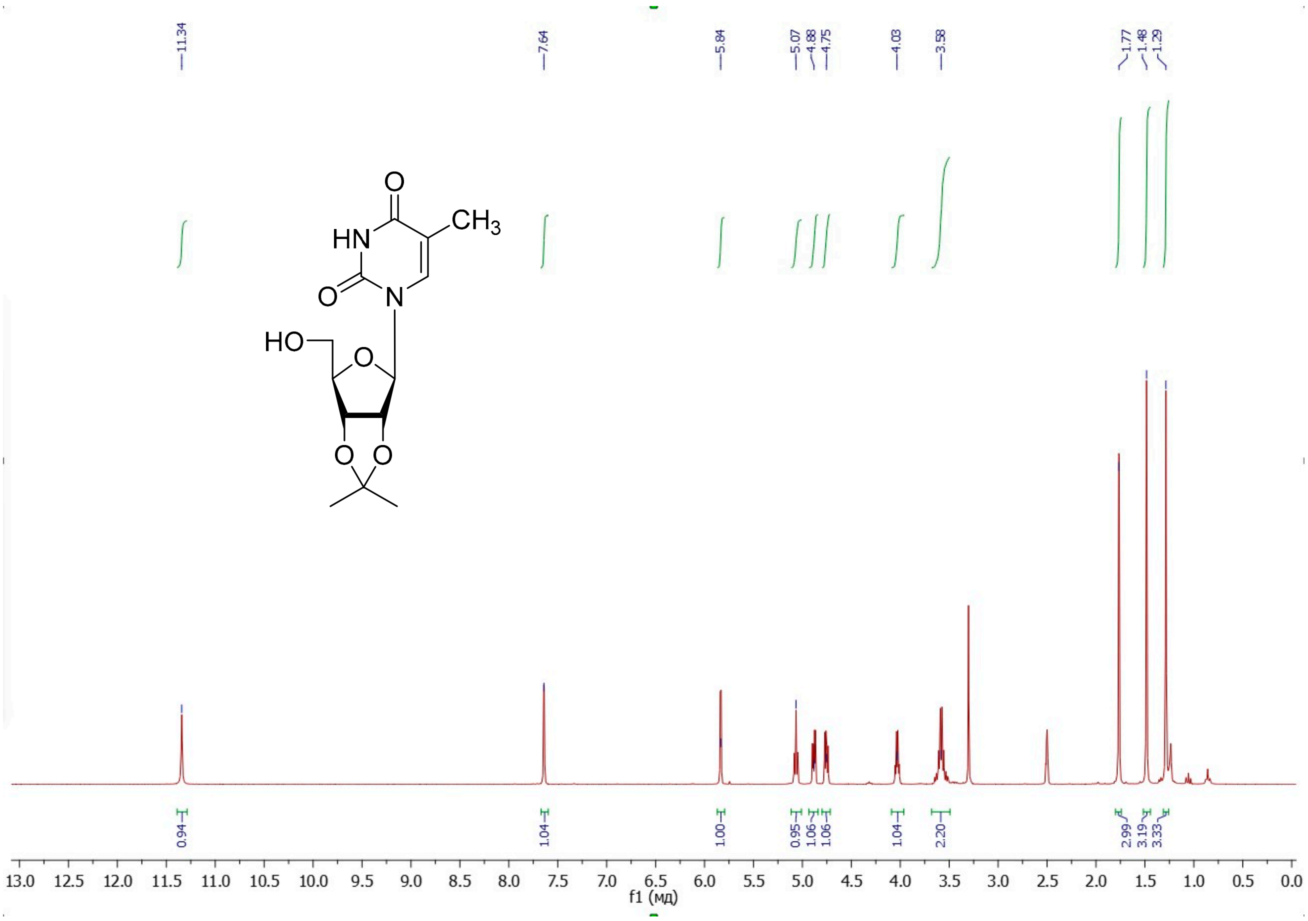

^{1}H-NMR-spectrum (300.1 MHz) of 2',3'-O-isopropylidene-5-methyluridine **(2b)** in DMSO-d_6 at 303 K

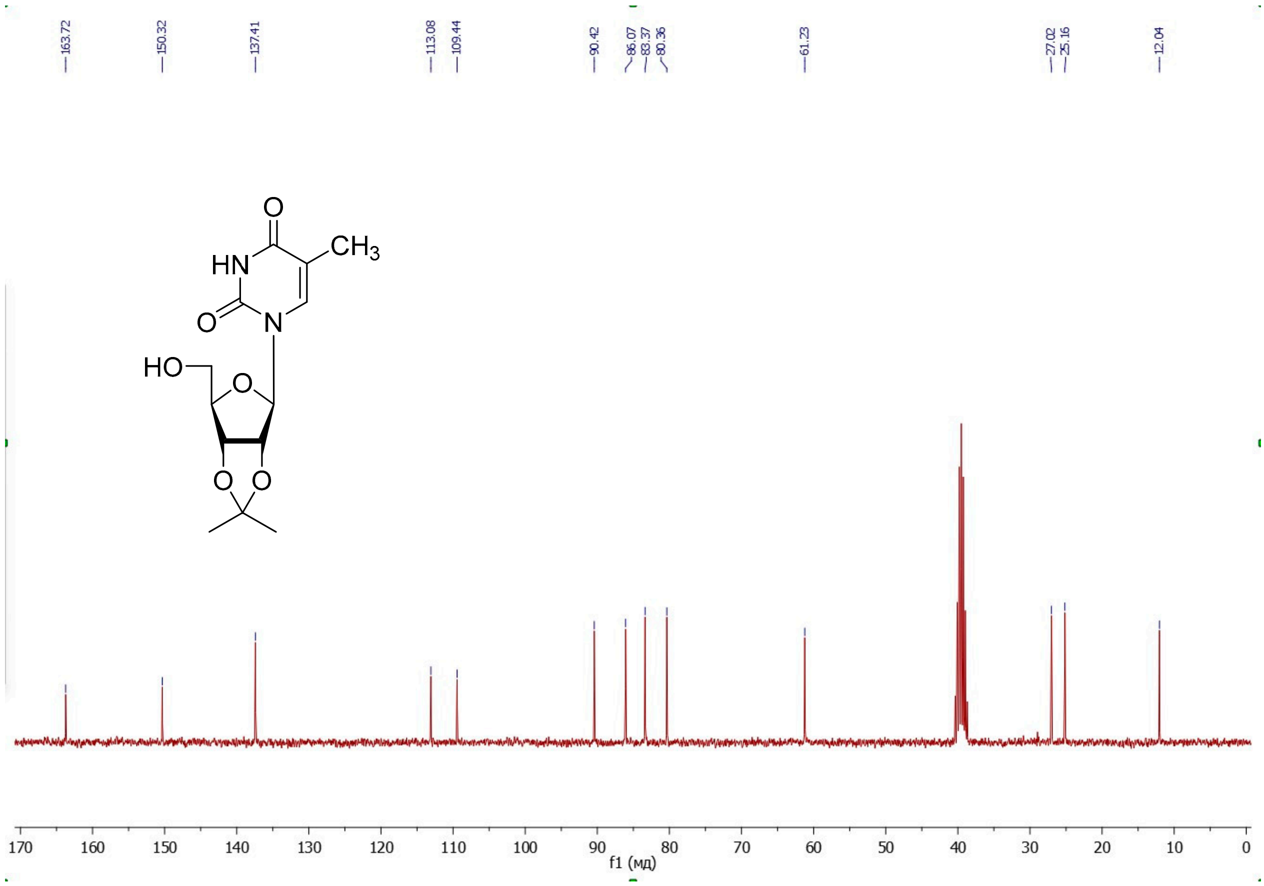

^{13}C-NMR-spectrum (75.5 MHz) of 2',3'-O-isopropylidene-5-methyluridine **(2b)** in DMSO-d_6 at 303 K

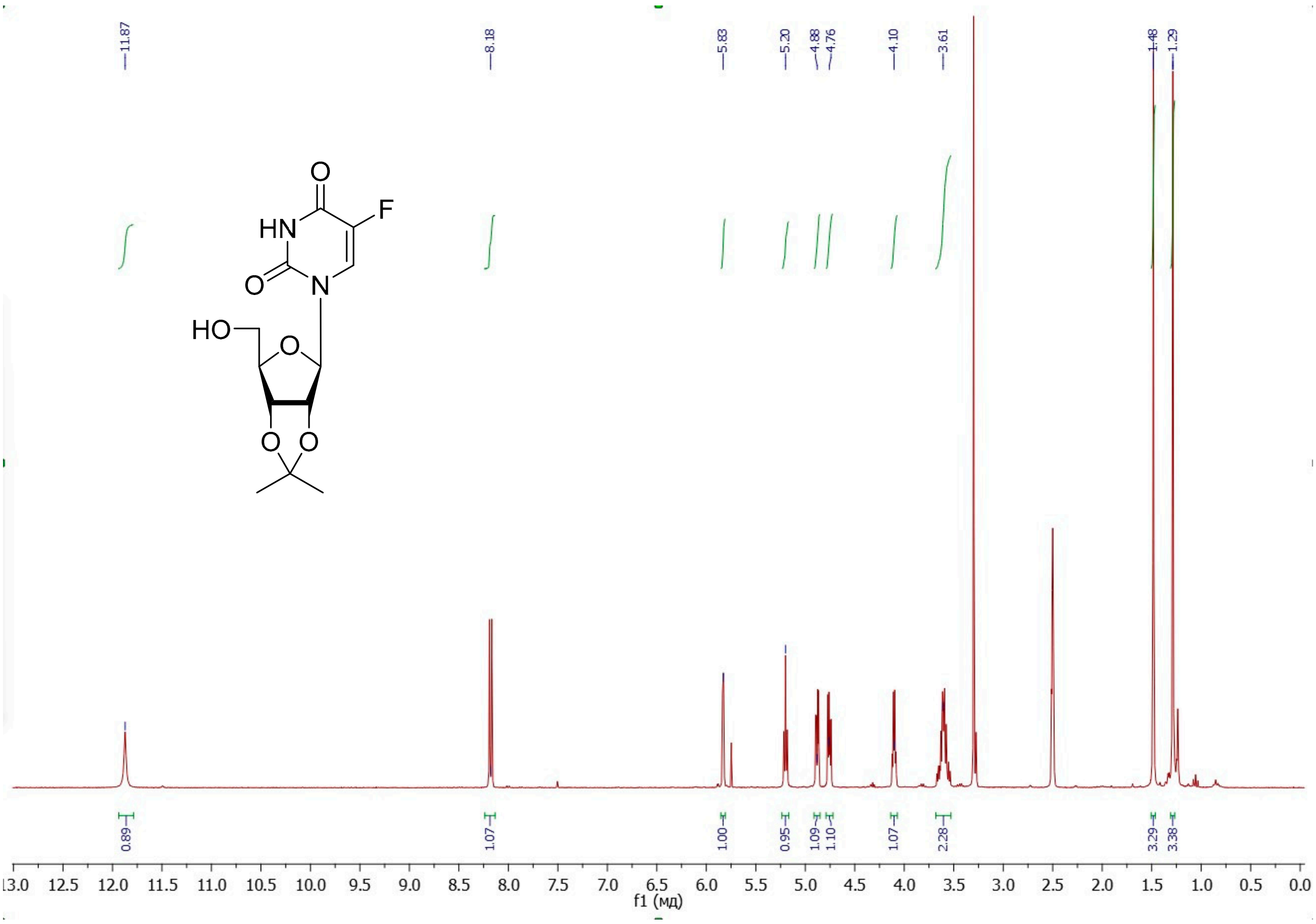

^{1}H-NMR-spectrum (300.1 MHz) of 2',3'-O-isopropylidene-5-fluorouridine **(2c)** in DMSO-d_6 at 303 K

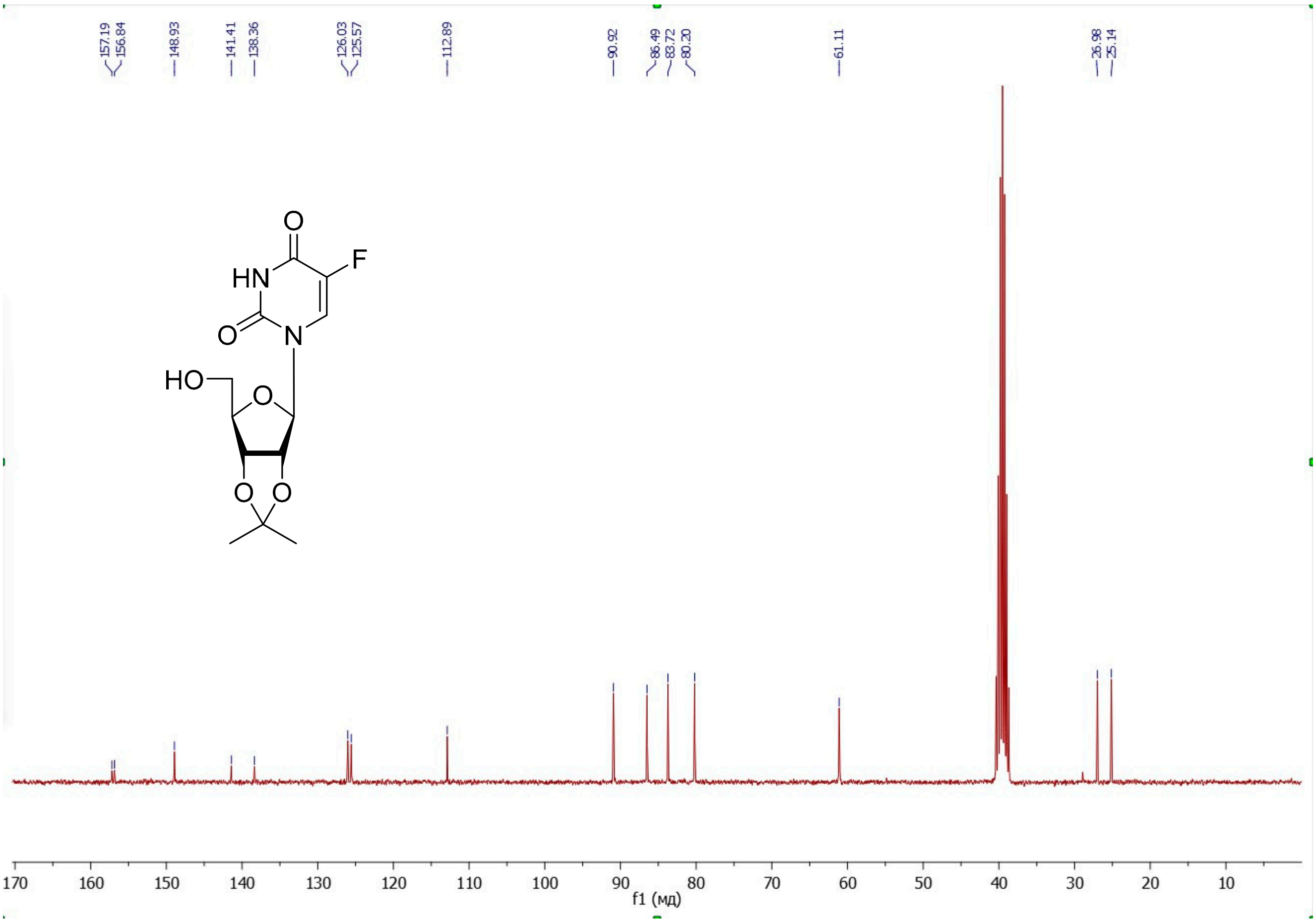

^{13}C-NMR-spectrum (75.5 MHz) of 2',3'-O-isopropylidene-5-fluorouridine **(2c)** in DMSO-d_6 at 303 K

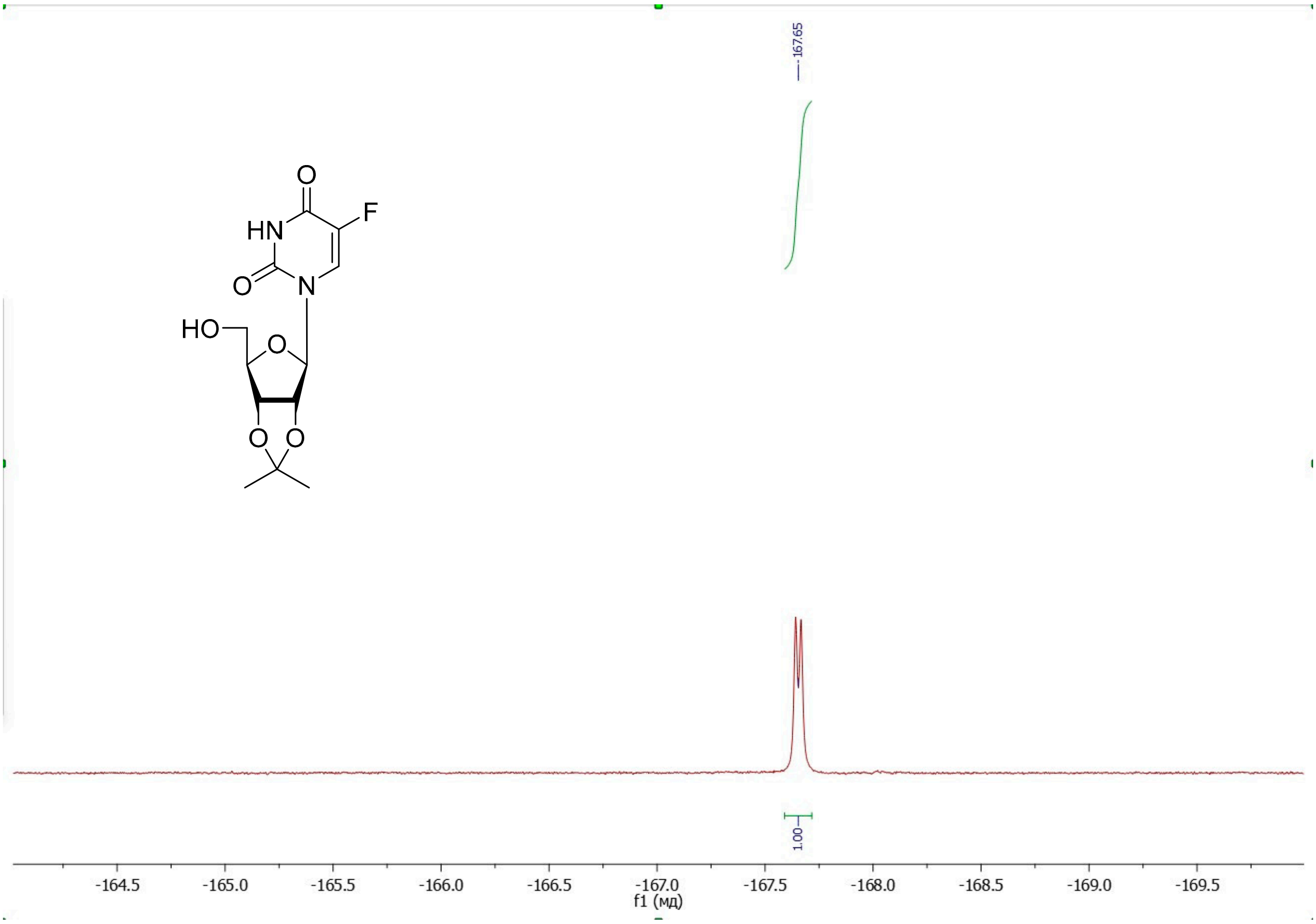

^{19}F-NMR-spectrum (282.4 MHz) of 2',3'-O-isopropylidene-5-fluorouridine **(2c)** in DMSO-d_6 at 303 K

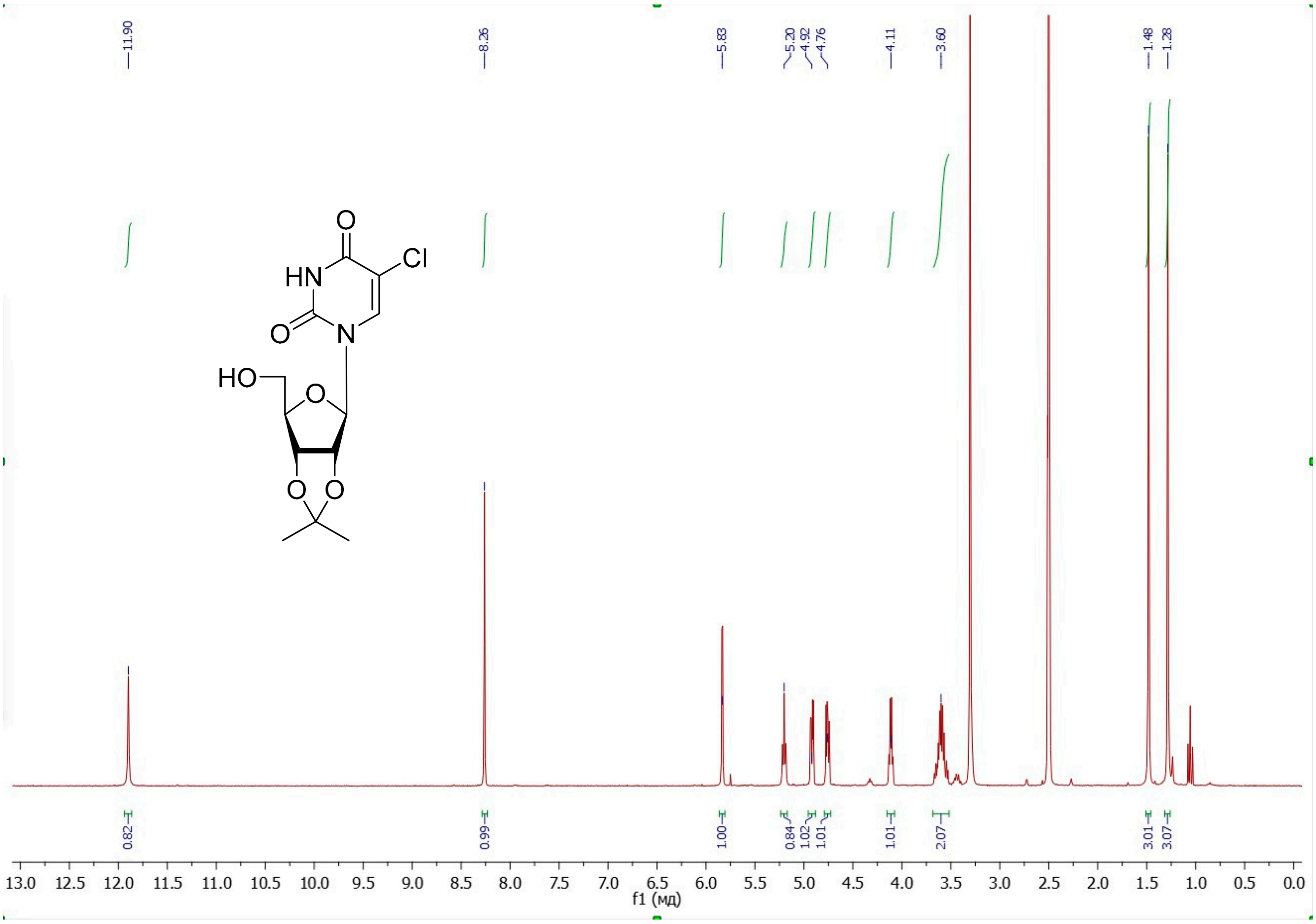

^{1}H-NMR-spectrum (300.1 MHz) of 2',3'-O-isopropylidene-5-chlorouridine **(2d)** in DMSO-d_6 at 303 K

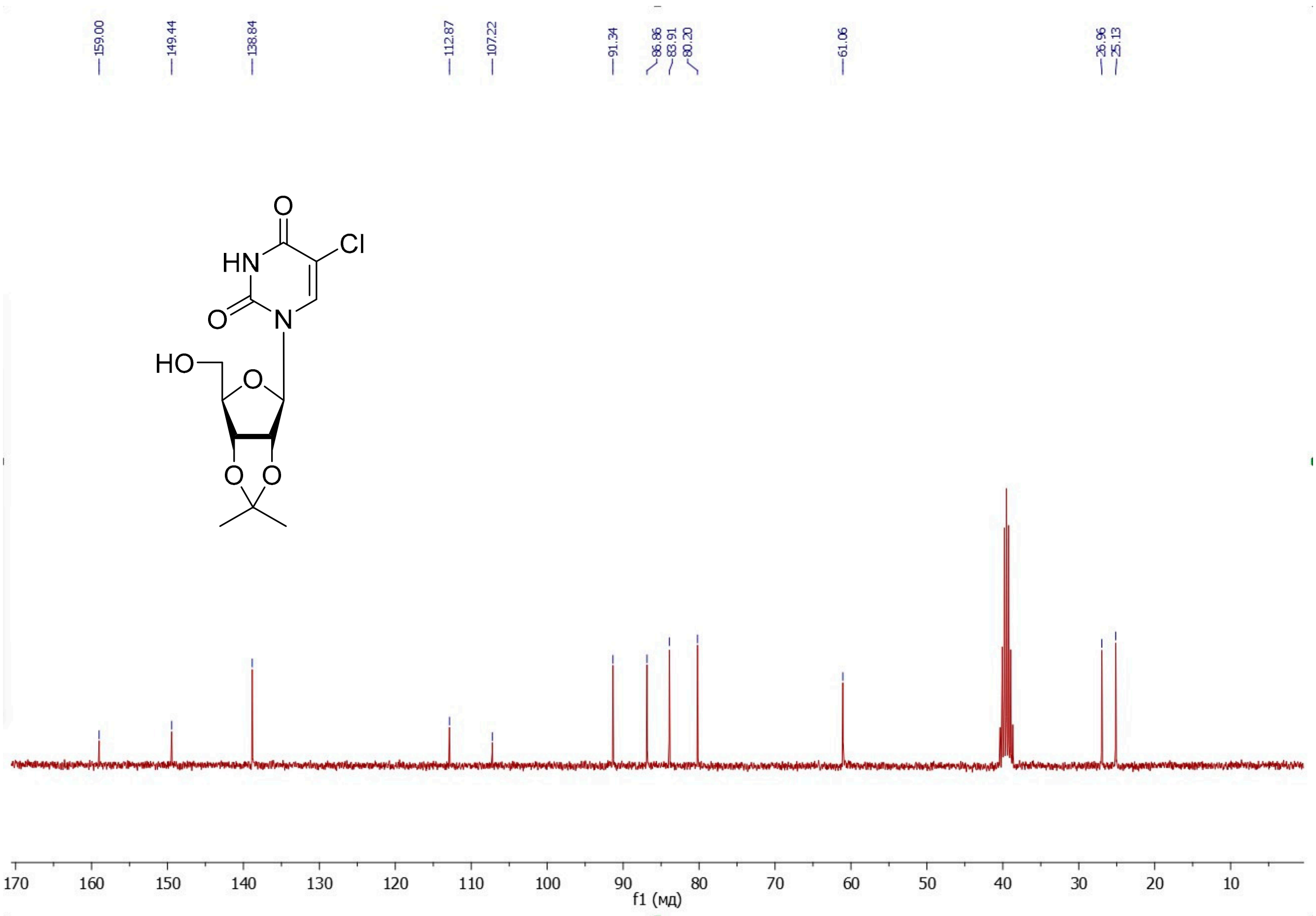

^{13}C-NMR-spectrum (75.5 MHz) of 2',3'-O-isopropylidene-5-chlorouridine **(2d)** in DMSO-d_6 at 303 K

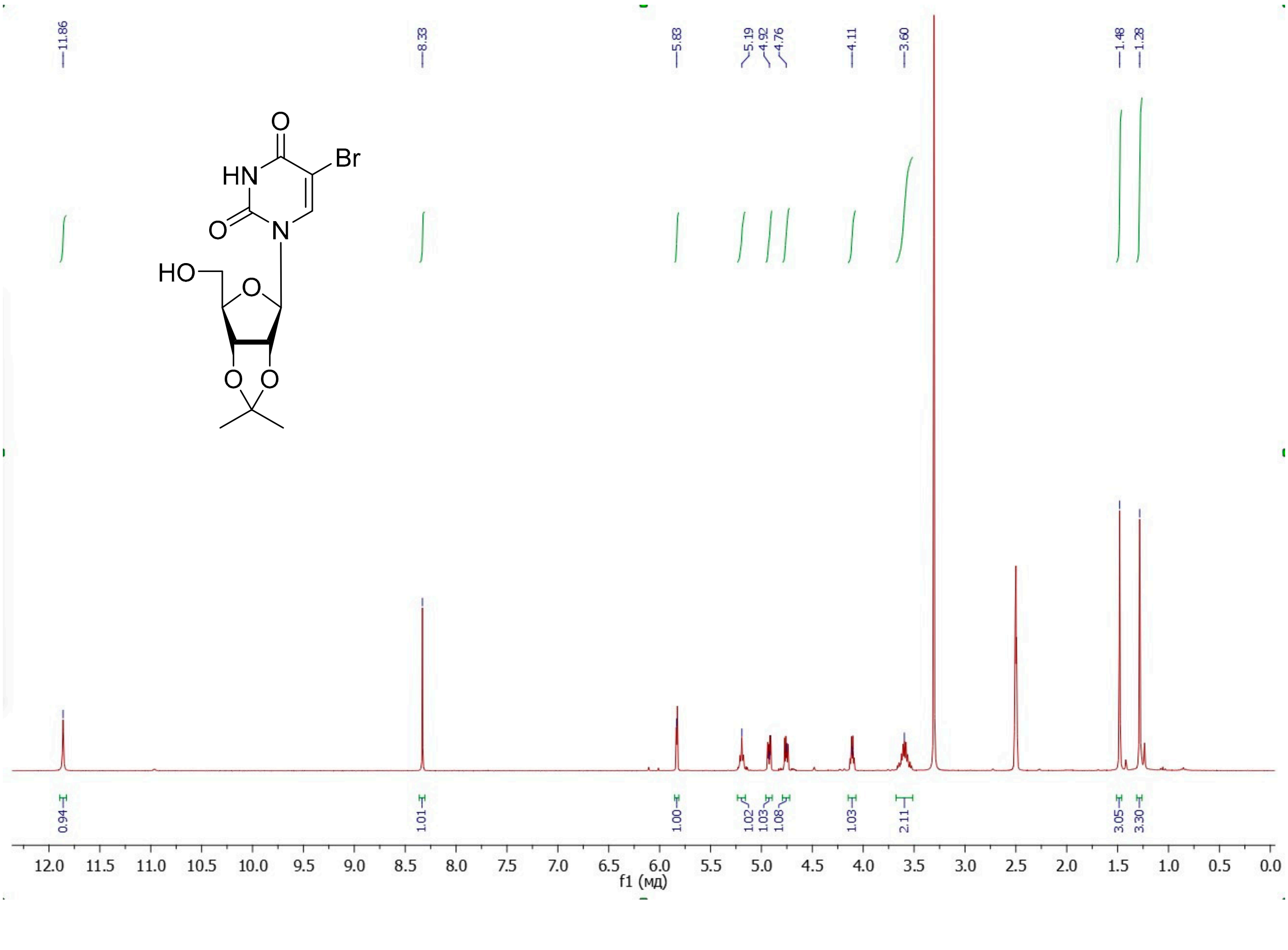

^{1}H-NMR-spectrum (300.1 MHz) of 2',3'-O-isopropylidene-5-bromouridine **(2e)** in DMSO-d_6 at 303 K

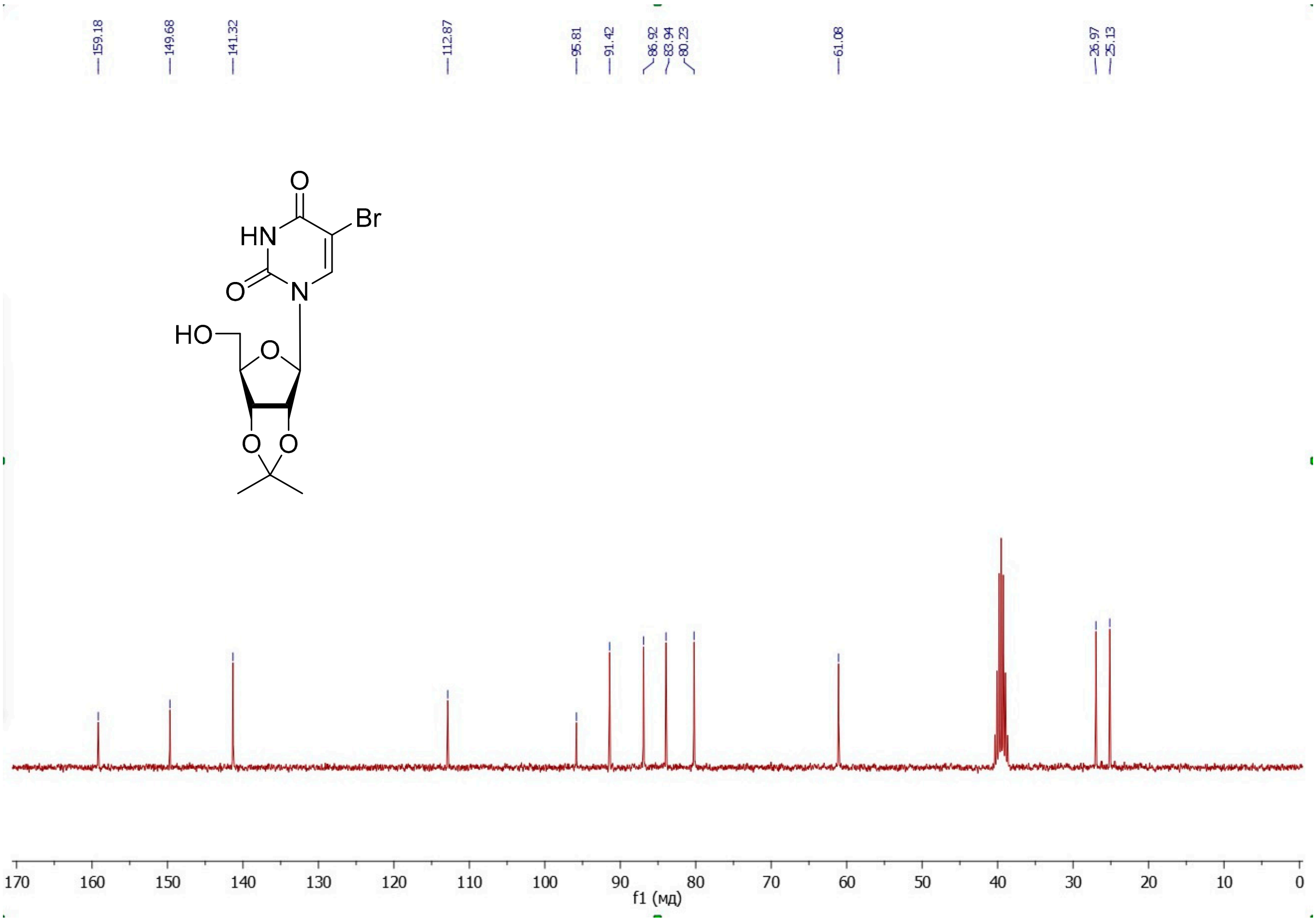

^{13}C-NMR-spectrum (75.5 MHz) of 2',3'-O-isopropylidene-5-bromouridine **(2e)** in DMSO-d_6 at 303 K

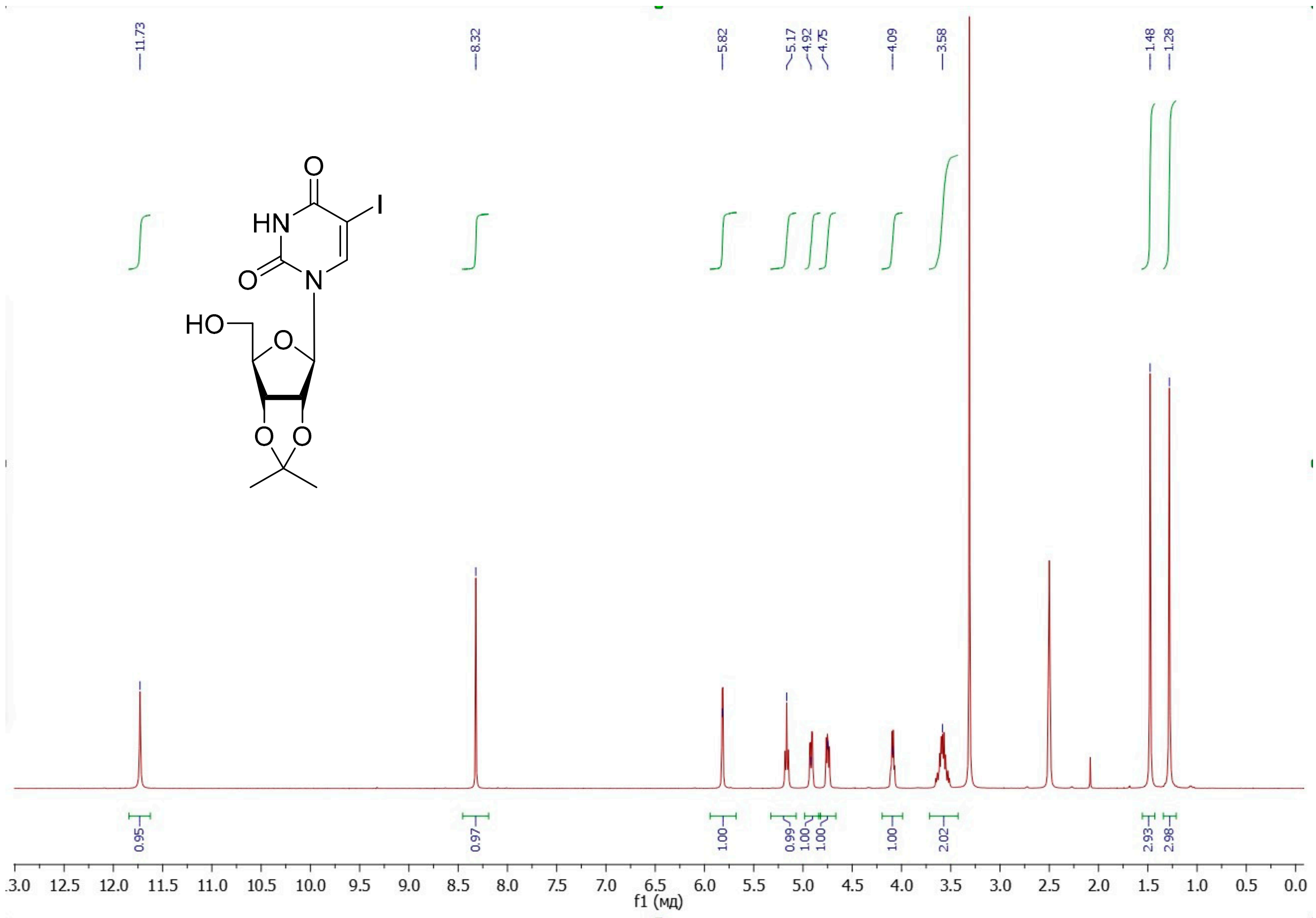

^{1}H-NMR-spectrum (300.1 MHz) of 2',3'-O-isopropylidene-5-ioduridine **(2f)** in DMSO-*d*₆ at 303 K

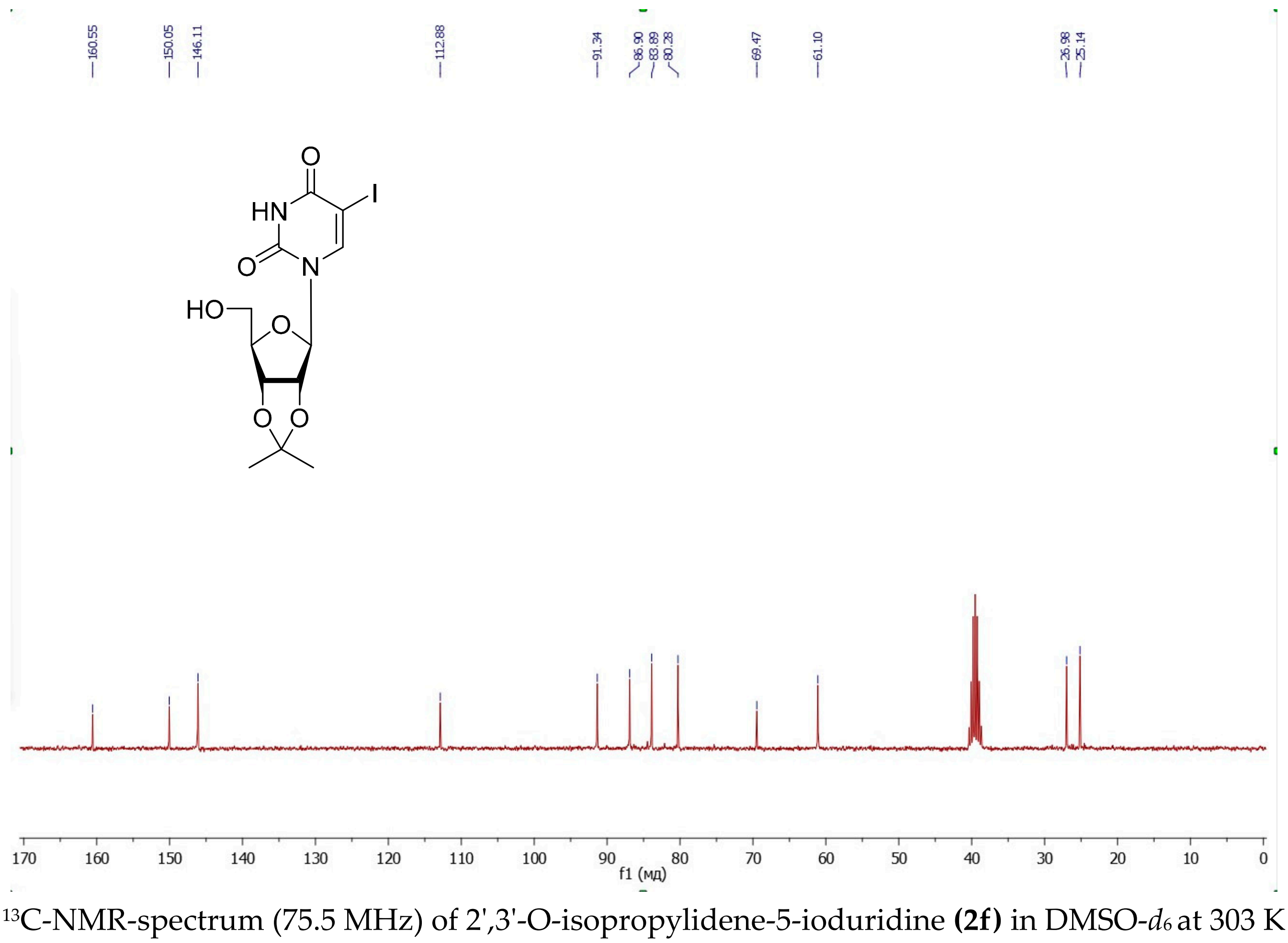

^{13}C-NMR-spectrum (75.5 MHz) of 2',3'-O-isopropylidene-5-ioduridine **(2f)** in DMSO-*d*₆ at 303 K

3. Mass-spectrometry

The mass spectra were acquired using a MALDI TOF mass spectrometer AB_SCIEX_4800Plus (AB Sciex, Framingham, MA, USA) equipped with an Nd:YAG laser in the linear mode. The analysis was performed using 2,5-dihydroxybenzoic acid as the matrix. Spectra were recorded for positive ions. The spectra contained both MH^+ peaks and $(M+Na)^+$ or $(M+K)^+$ peaks.

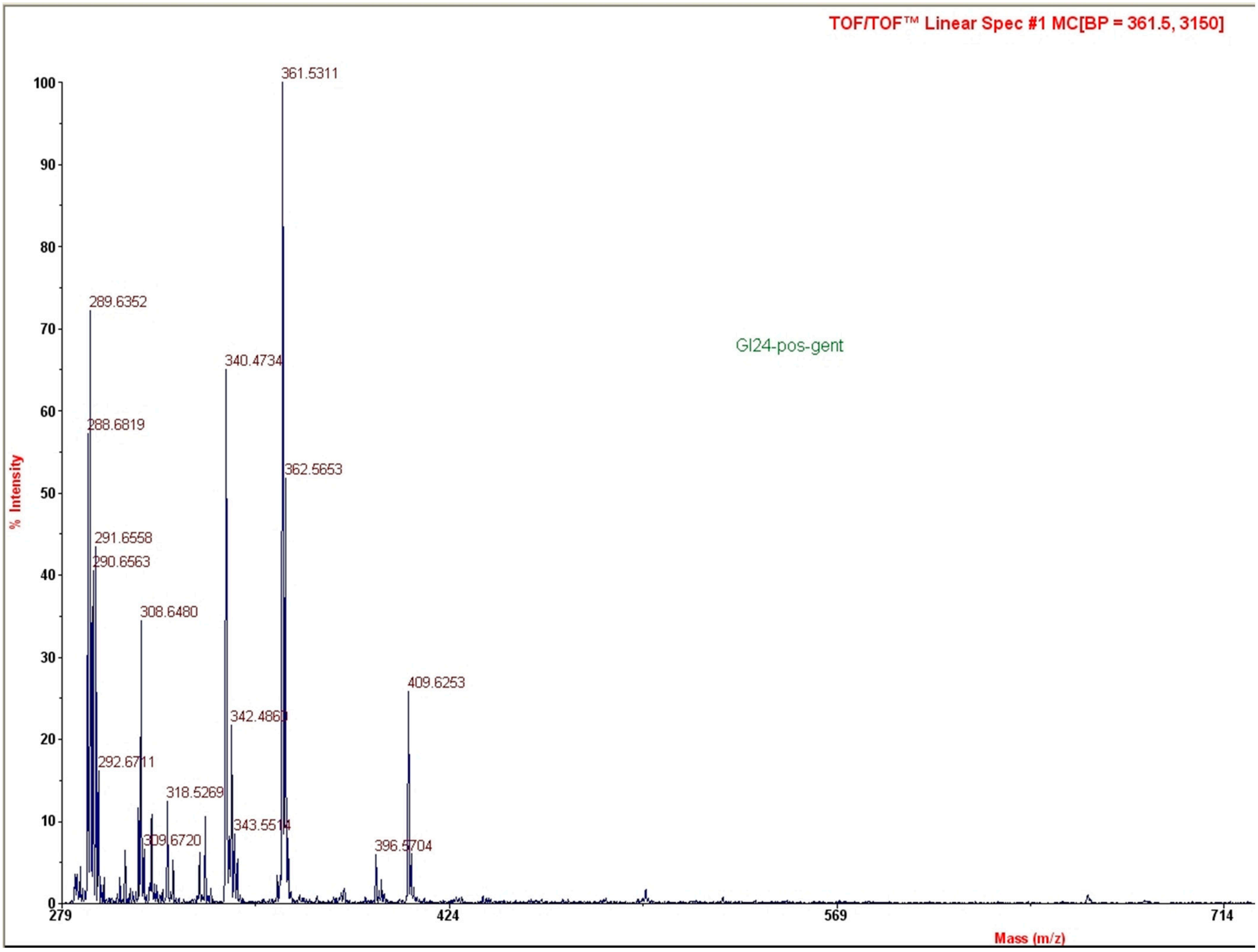

Mass spectrum (MALDI) of 2',3'-O-isopropylidene-5-ioduridine **(2f)**

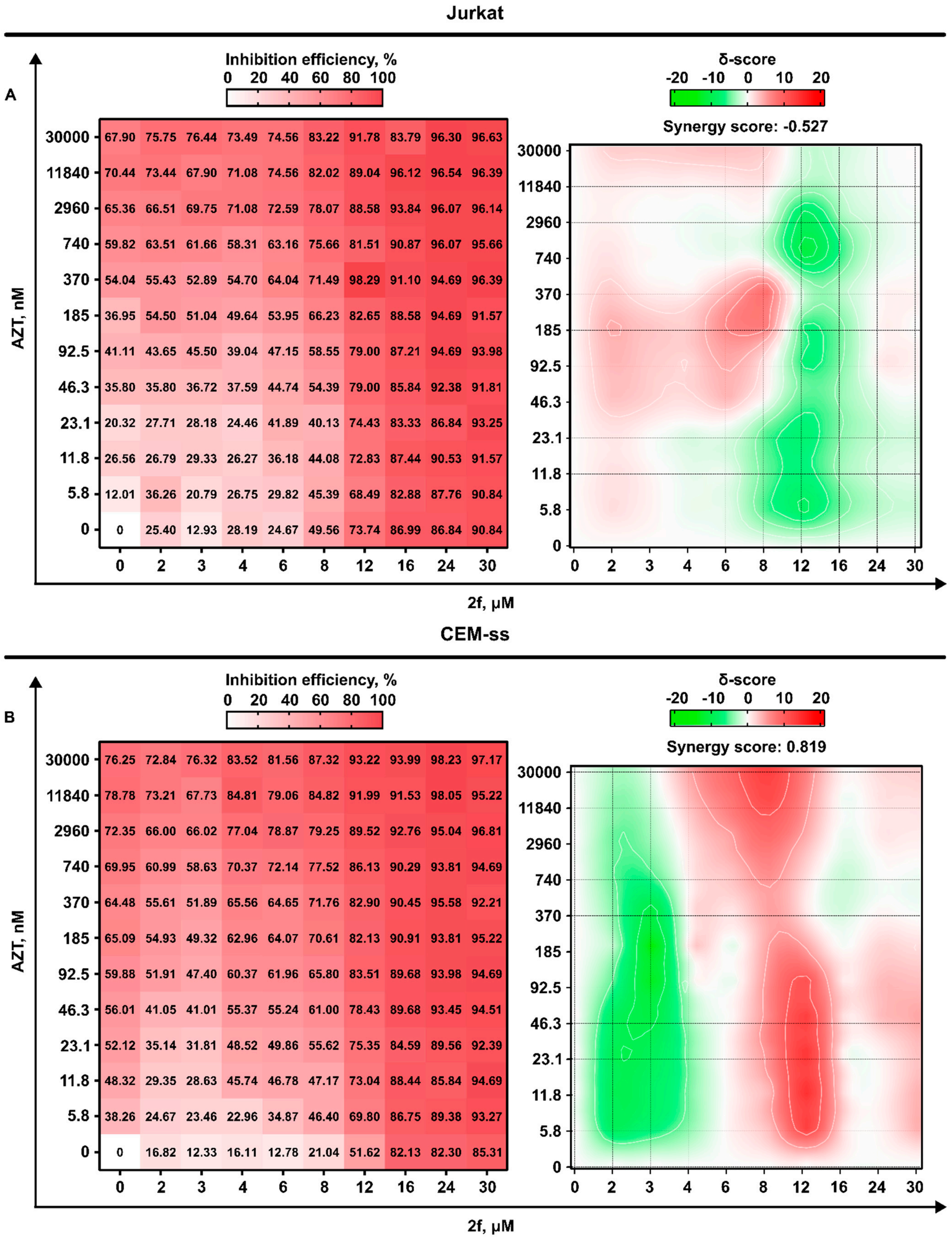

Figure S1. Analysis of the effect of 2f in combination with AZT. Jurkat and CEM-ss cells were treated with AZT and **2f** at the range of concentrations up to 30 μM. Cells were simultaneously treated with the inhibitors and transduced with VP-GFP. The action of the inhibitors on

transduction efficiency of VP-GFP 72 hours after transduction. **(A, B)** dose-response matrix represents the percentage of eGFP-negative cells (anti-viral effect) compared to non-treated cells. Synergy plots (left side of the Figure) represent the effect of the drug combination (synergism/additive effect/antagonism) calculated and visualized using Synergy Finder v3.0 software and a ZIP method. Red color highlight synergism, white — additive effect, green — antagonism.
